# Smart molecular design for functional cellulose gels and flexible devices

**DOI:** 10.1002/smo2.70038

**Published:** 2026-02-05

**Authors:** Zeshi Li, Minxin Wang, Geyuan Jiang, Jianhong Zhou, Donghan Li, Weihua Zhang, Dawei Zhao

**Affiliations:** ^1^ Key Laboratory on Resources Chemicals and Materials of Ministry of Education Shenyang University of Chemical Technology Shenyang China; ^2^ College of Biomass Science and Engineering & Key Laboratory of Leather Chemistry and Engineering Ministry of Education Sichuan University Chengdu China; ^3^ State Key Laboratory of Woody Oil Resources Utilization Key Laboratory of Bio‐Based Material Science and Technology of Ministry of Education Northeast Forestry University Harbin China

**Keywords:** cellulose, flexible electronics, flexible robotics, functional gels, reinforcement design

## Abstract

Cellulose, the dominant natural polymer on Earth, features a distinct molecular structure with extraordinary mechanical properties and tunable characteristics, making it attractive for gel systems. Although significant progress has been made, challenges remain in fully leveraging their functional potential and broadening practical applications. This review systematically examines the properties of cellulose and cellulose gels, exploring novel reinforcement strategies—across molecular, supramolecular network, and macroscale structure levels—to enhance mechanical, electrical, and thermal performance, while coordinating these properties for practical implementations. These advancements are exemplified in emerging fields such as flexible robotics, electronic skins, flexible energy storage devices, and human‐machine interaction systems. This article thoroughly investigates the fundamental characteristics, multi‐scale design approaches, performance enhancement mechanisms, and cutting‐edge implementations of cellulose‐based gels across diverse domains. It provides a comprehensive overview of these advanced materials and offers strategic insights and recommendations for future research and innovation.

## Graphical abstract

This review systematically outlines the performance enhancement of cellulose gels, presenting optimization strategies across molecular, supramolecular, and macroscopic scales. It discusses advanced approaches to improve their mechanical, electrical, and thermal properties, and highlights promising applications in flexible robotics, electric devices, e‐skins, and human‐machine interaction. This paper offers valuable perspectives and future research directions for the design of cellulose gels.

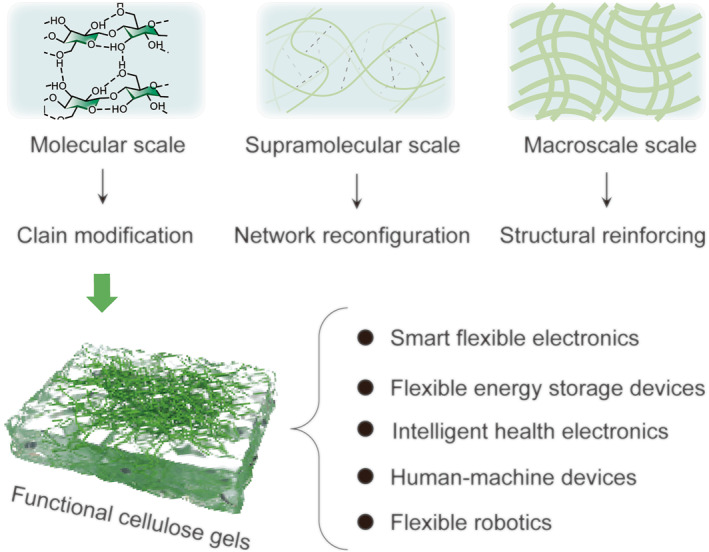

## 1 INTRODUCTION

The global dependence on conventional fossil fuels has precipitated a significant energy crisis and exacerbated environmental degradation, underscoring the necessity of transitioning to sustainable and renewable energy sources and materials. Biomass resources, such as chitosan, hemicellulose, and lignin, are promising alternatives owing to their renewable nature, abundant supply, and potential for carbon neutrality. Among them, cellulose is recognized as the most abundant natural polymer on Earth. Its significance stems not only from its renewability but also from its distinct hierarchical structure, which spans from the molecular to the macroscopic scale. This multi‐level architecture, rich in hydroxyl groups, facilitates extensive hydrogen bonding, thereby conferring remarkable mechanical strength, biodegradability, and biocompatibility.^[1]^ Consequently, cellulose serves as more than a mere substitute for non‐renewable resources, thereby​ providing an adaptable basis for developing advanced materials capable of reducing the ecological impacts linked to fossil fuel consumption.

The 19th‐century witnessed the emergence of gel research marked by the precise definition of the term “gel” and its fundamental properties. This development was primarily driven by the demand for materials capable of immobilizing large volumes of liquid within a solid‐like three‐dimensional network. This cross‐linked structure provides gels with key properties, such as high absorbency, environmental responsiveness, and tunable mechanical strength.^[2]^ These properties have driven their adoption in diverse fields and continue to propel the expansion of gel applications, particularly as sustainable and intelligent materials in biomedical engineering,[^3^, ^4^] flexible electronics, and environmental remediation. The expanding applications of gels have intensified the search for ideal materials. In this context, cellulose has emerged as a prominent research focus, owing to its inherent advantages such as renewability, biodegradability, and a high density of hydroxyl groups on its molecular chains. The hydroxyl groups facilitate strong yet adjustable intermolecular interactions (e.g., hydrogen bonding), directly enabling beneficial characteristics in cellulose gels, including high water retention, significant adsorption capacity, biocompatibility, and tunable mechanical properties. Thus, cellulose gels show strong potential for applications in flexible electronics (e.g., as substrates or sensors), biomedical devices (e.g., drug delivery or tissue engineering), environmental remediation, and advanced agricultural products.[^5^, ^6^, ^7^, ^8^, ^9^, ^10^, ^11^, ^12^, ^13^]

However, despite these advantages, cellulose gels often face the challenge of balancing mechanical strength, electrical conductivity, and thermal stability, which is crucial for demanding real‐world applications where these properties must function synergistically. To overcome these limitations and achieve the required performance balance, intentional design strategies​ that enable coordinated enhancement​ of multiple properties are essential. This review systematically summarizes the recent advances in performance enhancement strategies for cellulose gels in mechanical strength, electrical conductivity, and thermal stability. It elaborates on multi‐scale design approaches, ranging from molecular‐level engineering to supramolecular network construction, and further to macro‐scale structural design. Subsequently, the review highlights the cutting‐edge applications of these advanced cellulose gels, particularly in flexible robotics as actuators and grippers, and in flexible electronics such as e‐skins, supercapacitors and batteries. The review further explores the application potential of these advanced cellulose gels in human‐machine interfaces (HMI) and AI‐deep learning systems, highlighting their promise within the current wave of artificial intelligence development. The overarching goal is to offer a comprehensive perspective on how rational design at different scales can enhance the performance of cellulose gels, thereby accelerating their application in next‐generation intelligent devices.

## 2 CELLULOSE AND FUNCTIONAL GELS

### 2.1 Properties and molecular structure of cellulose

**FIGURE 1:**
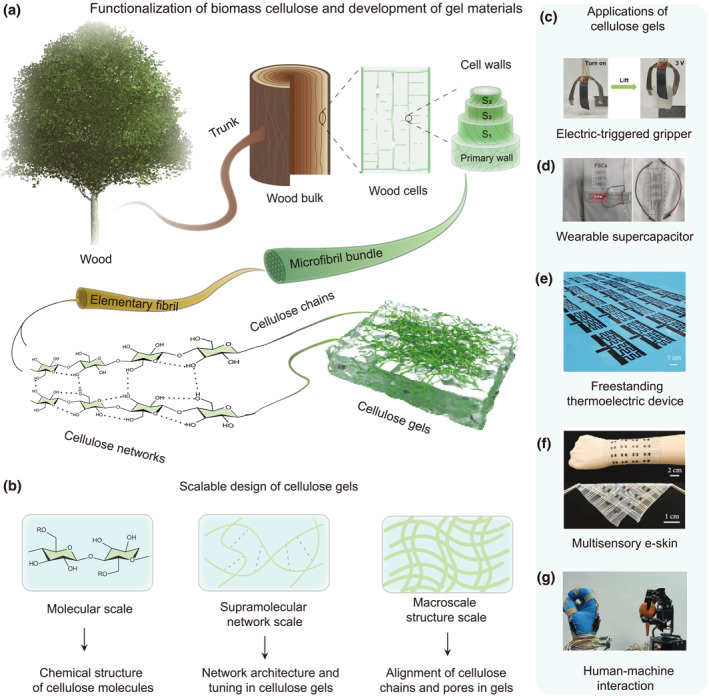
Fabrication, design levels, and applications of cellulose gels. (a) Schematic illustration of cellulose natural sources and fabrication of cellulose gels. (b) Schematic illustration of design levels of cellulose gels. (c–g) Photos of novel applications of cellulose gels: (c) Electric‐triggered gripper. Reproduced with permission.^[14]^ Copyright 2025, Wiley‐VCH. (d) Wearable supercapacitor. Reproduced with permission.^[15]^ Copyright 2025, Elsevier. (e) Freestanding thermoelectric device. Reproduced with permission.^[16]^ Copyright 2025, Wiley‐VCH. (f) Multisensory e‐skin. Reproduced with permission.^[17]^ Copyright 2022, AAAS. (g) Human‐machine interaction. Reproduced with permission.^[18]^ Copyright 2024, Wiley‐VCH.

Cellulose, a natural polymer sourced from plants like trees, cotton, and bamboo, as well as from certain bacteria and algae, possesses a linear chain structure of β‐1,4‐linked D‐glucose units. Abundant hydroxyl groups along these chains facilitate extensive hydrogen bonding, endowing cellulose with high tensile strength, stiffness, and flexibility. This molecular architecture is organized hierarchically, from elementary fibrils to macro‐scale fibers, enabling engineering into diverse morphologies such as films, fibers, and gels (Figure 1). This multi‐level organization allows for precise tuning of properties such as porosity, specific surface area, and thermal stability.^[19]^

The extraction of cellulose typically involves chemical pretreatments to remove lignin and hemicellulose, followed by dissolution using green solvents that disrupt the hydrogen‐bond network, such as ionic liquids (ILs) or alkali/urea systems.[^20^, ^21^] These processing methods leverage cellulose's inherent advantages, including its ability to form robust three‐dimensional networks, its high adsorption capacity, and its ease of chemical modification. These characteristics, combined with sustainable processability, make cellulose an ideal substrate for advanced applications in flexible electronics, energy storage, and biomedical implants, where performance and ecological sustainability are paramount. Furthermore, the dynamic nature of hydrogen bonding allows material properties to be regulated by solvents or external stimuli, facilitating the design of adaptive, smart materials and underscoring cellulose's critical role in next‐generation sustainable technologies.

### 2.2 Fabrication of functional cellulose gels

**FIGURE 2:**
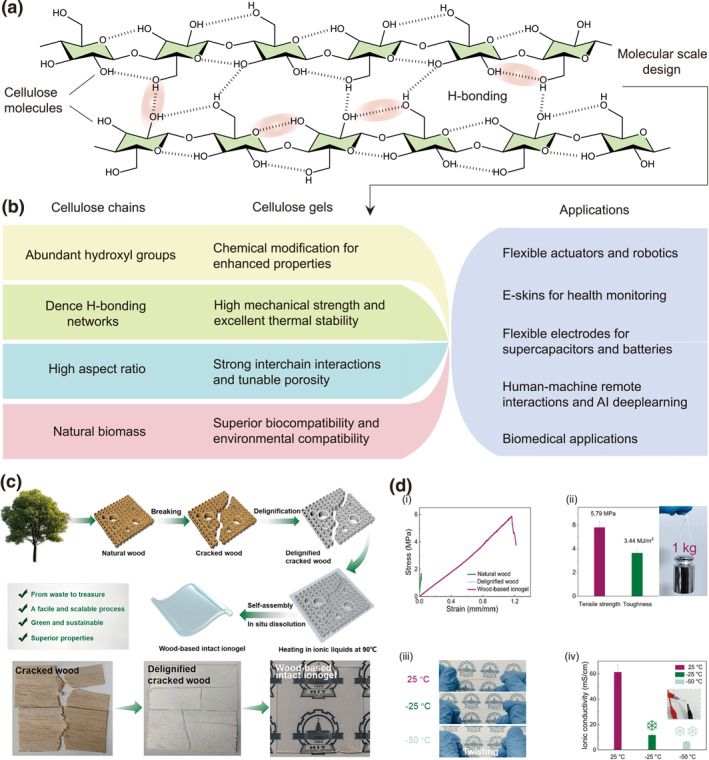
Properties of cellulose gels. (a) Schematic illustration of cellulose chains and the dense hydrogen bonding networks from innerchain and interchain. (b) Properties of cellulose chains, cellulose gels and advanced applications of cellulose gels. (c, d) All‐cellulose ionogel. (c) Schematic diagram and photos of the fabrication of all‐cellulose ionogels. (d) Properties of all‐cellulose ionogels. (i) Tensile stress–strain curves of natural wood, delignified wood, and all‐cellulose ionogel. (ii) Elastic modulus and fracture toughness, and photo proving toughness. (iii) Digital images showing the appearance and twisting at different temperatures. (iv) Ionic conductivities at different temperatures. Reproduced with permission.^[22]^ Copyright 2024, Elsevier.

Cellulose gels inherit their versatile functionality directly from the intrinsic properties of the parent cellulose polymer. Specifically, its robust hydrogen‐bonding capability enables the formation of a three‐dimensional network primarily through physical cross‐linking (Figure 2a), while the abundance of modifiable hydroxyl groups allows for chemical cross‐linking or functionalization. The high aspect ratio of cellulose nanofibrils further contributes to the gel's high specific surface area.​ This synergy enables precise control over the nanoscale architecture, primarily the porosity and pore‐size distribution. The inherent biocompatibility of native cellulose is also preserved, paving the way for the development and application of these gels in the biomedical field (Figure 2b).

This controlled structural architecture is key to the exceptional and tunable properties of cellulose gels. Mechanically, robustness arises from a synergy between a rigid nanofiber scaffold and the dynamic hydrogen‐bonding network, which dissipates energy under load and facilitates self‐healing, thereby enhancing durability. Electrically, as exemplified by cellulose ionogels, the incorporation of ILs creates efficient ion transport pathways, yielding high ionic conductivity crucial for flexible electronics. The selection of IL anions and cations further allows for precise regulation of both conductivity and mechanical properties.^[23]^

A compelling example is a biodegradable all‐cellulose ionogel produced from low‐value wood via a sustainable route involving delignification and in situ dissolution (Figure 2c).^[22]^​ The resulting material exhibits exceptional mechanical strength, approaching 6 MPa with an elongation exceeding 115%, due to a reconstituted hydrogen‐bonding network. The ionogel exhibits excellent electrical performance over a wide temperature range. It achieves a high ionic conductivity of approximately 60 mS/cm at 25°C, which remains well‐maintained at 6.4 mS cm^−1^ even at −50°C. This performance stems from continuous ion pathways and dynamic physical crosslinks (Figure 2d).

The “1 + 1>2” synergistic design principle is a common and effective strategy in gel engineering, where cellulose is frequently incorporated as a robust structural scaffold to significantly enhance mechanical strength and durability. This framework is then synergistically combined with conductive components which provide efficient pathways for electron or ion transport. This classic design approach successfully marries high mechanical resilience with excellent electrical conductivity, creating composite materials whose overall performance surpasses the simple sum of their individual parts.[^24^, ^25^, ^26^] Consequently, it serves as a versatile and reliable blueprint for developing advanced functional materials, particularly for applications in flexible electronics where both structural integrity and conductive performance are critical.

Benefiting from this combination of biocompatibility, tunable mechanical/electrical properties, self‐healing ability, and sustainability, cellulose gels show broad application prospects. In biomedicine, they serve as wound dressings, drug carriers, and tissue engineering scaffolds. For instance, one study developed a multifunctional cellulose nanofiber‐reinforced hydrogel that combines antimicrobial, antioxidant, self‐healing, and adhesive properties, showing considerable potential as advanced wound dressing.^[27]^ Meanwhile, another study designed a near‐infrared‐responsive carboxymethyl cellulose hydrogel incorporating thioketal linkages, which enables precise on‐demand drug release for controlled delivery applications.^[28]^ In flexible electronics, they are suitable for supercapacitors, sensors, and electronic skins. Their high adsorption capacity and thermal insulation properties also enable applications in water treatment and thermal management. Through sophisticated network design, cellulose gels thus provide a versatile platform for next‐generation technologies that harmonize high performance with ecological sustainability. To fully unlock these potentials, the synergistic balance of mechanical resilience, electrical conductivity, and thermal stability is paramount.​ This integrated performance enables cellulose gels to function reliably in complex real‐world applications, thereby driving their transition from versatile platforms to practical solutions.

## 3 MECHANICAL STRATEGIES FOR FUNCTIONAL GELS

While cellulose gels inherently possess commendable mechanical strength, biocompatibility, and renewability, limitations in achieving the synergistic combination of high strength, exceptional toughness, and tunable or customized functionality still restrict them from advanced applications. These intrinsic shortcomings necessitate the development of sophisticated mechanical reinforcement strategies to precisely engineer their performance across multiple length scales.[^29^, ^30^] The primary objective is to integrate effective energy dissipation mechanisms and enhance the load‐bearing capacity without compromising the material's sustainable attributes or processability. The design principles for augmenting the mechanical properties of cellulose gels are focused on a hierarchical approach spanning from molecular functionalization to macroscopic structuring. The discussion is organized into three key levels: molecular‐level strategies leveraging chemical modification of functional groups, supramolecular network‐level tactics utilizing tunable physical interactions, and macroscale structure‐level methodologies employing alignment and hierarchical design. Each level offers distinct mechanisms to control chain packing, intermolecular bonding, and stress distribution, collectively advancing the frontier of robust and functional cellulose gel materials.

### 3.1 Molecular strategies: Chemical modification of functional groups

Chemical modification, leveraging the abundant hydroxyl groups on cellulose, is a powerful strategy to tailor molecular structures and overcome inherent material limitations. By introducing diverse functional groups (e.g., carboxymethyl group,^[31]^ cyano group,^[32]^ sulfonic group,^[33]^ phosphate ester group^[34]^), these modifications not only enhance mechanical properties—such as strength, stretchability, and toughness—but also preserve cellulose's renewable and biodegradable advantages, often adding novel functionalities like ionic conductivity or thermoelectricity.

**FIGURE 3:**
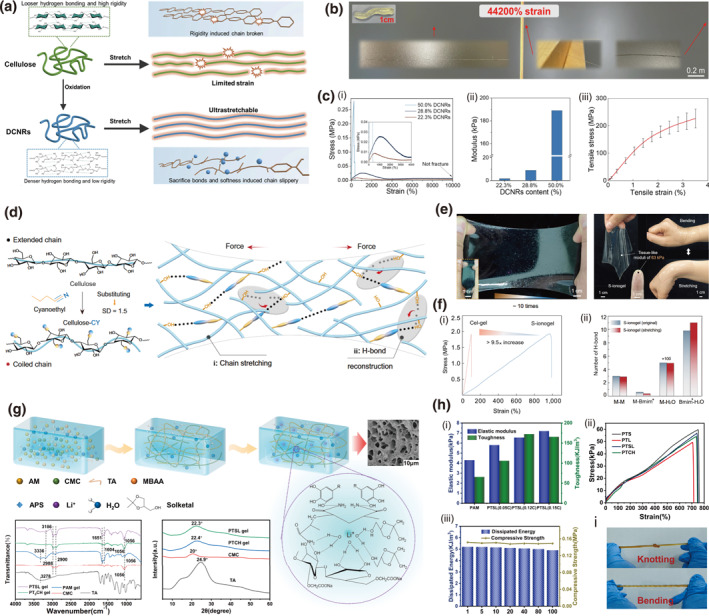
Chemical modification of cellulose gels. (a–c) Ring opening modification on cellulose: (a) Schematic illustration showing the ring‐opening of cellulose and comparison between cellulose and modified DCNRs. (b) Optical images of DCNRs hydrogel before and after stretching. (c) Mechanical performance of DCNR gel: (i) Stress–strain curves under different DCNR contents and (ii) the corresponding modulus. (iii) Average stress–strain curve of dried DCNR fiber. Reproduced with permission.^[36]^ Copyright 2024, Elsevier. (d–f) Cyanoethylation of cellulose: (d) Schematic illustration showing the cyanoethylation modification of cellulose and comparison between cellulose and stretchable molecular networks of S‐ionogel. (e) Optical images of the S‐ionogel exhibiting flexibility, tissue resilience, and seamless integration. (f) Mechanical performance and reinforcement mechanism of S‐ionogel: (i) Tensile stress–strain curves of our S‐ionogel and traditional Cel‐gel. (ii) Comparing the number of H‐bonds in S‐ionogel before and after stretching. Reproduced with permission.^[37]^ Copyright 2024, AAAS. (g–i) Carboxymethylation of cellulose: (g): Diagrammatic illustration of the PTSL hydrogel's internal connections and manufacturing process, and SEM, FTIR, XRD characterizations of PTSL hydrogel. (h) Comparison of Modulus of Elasticity and Toughness of PAM and PTSL prepared at different mass. (i) Optical images of knotting and bending of the PTSL hydrogel. Reproduced with permission.^[38]^ Copyright 2024, Elsevier.

As a specific example, the periodate oxidation/borohydride reduction strategy linearizes rigid cellulose chains by opening pyranose rings, converting secondary to primary hydroxyls. This enhances chain flexibility and drastically increases the potential for forming dynamic, reversible hydrogen bonds, which are key to achieving superior mechanical properties.^[35]^ A highly stretchable all‐cellulose hydrogel is fabricated through this two‐step modification, producing dialcohol cellulose nanorods (DCNRs).^[36]^ The abundant primary hydroxyls on DCNRs facilitate the formation of a rigid hydrogen‐bonding network (Figure 3a), endowing the hydrogel with ultrahigh stretchability and rapid self‐healing capabilities. Furthermore, stretching the gel to its remarkable maximum strain exceeding 44,200% readily yields a robust cellulose‐based fiber (Figure 3b). At a DCNR content of 28.8%, the hydrogel demonstrates optimal tensile properties, achieving a tensile stress of 25 kPa and a modulus of 9 kPa. This fiber, derived from the aligned network, exhibits high tensile strength (up to 226 MPa) and modulus (9.4 GPa) (Figure 3c), making it suitable for practical applications.

The cyanoethylation strategy represents a chemical modification approach where hydroxyl groups on cellulose are partially substituted with cyanoethyl groups. This substitution induces a transformation from an extended to a coiled chain configuration, a design inspired by natural rubber that overcomes the intrinsic rigidity of cellulose and facilitates the formation of a dynamic hydrogen‐bonding network, which is crucial for enhancing mechanical properties. A cyano modified cellulose ionogel exhibits remarkable mechanical performance (Figure 3d), with high stretchability of exceeding 1000% strain (Figure 3e) and tensile strength of 1.78 MPa and skin‐like modulus of 63 kPa (Figure 3f).^[37]^ This performance effectively addresses the typical trade‐off between extensibility and robustness in material design. Furthermore, the ionogel demonstrates multifunctionality characterized by a notable Seebeck coefficient of approximately 68 mV K^−1^, indicating its thermoelectric capability, along with favorable biocompatibility. These attributes establish the material as a promising candidate for applications in self‐powered flexible electronics, such as electronic skins (e‐skins).

Carboxymethyl cellulose (CMC) is synthesized through the carboxymethylation of natural cellulose, a process that enhances its hydrophilicity and solubility by introducing carboxylmethyl groups. This derivative provides notable advantages including biocompatibility, tunable physicochemical properties, and ease of functionalization, making it highly suitable for advanced material applications.^[39]^ A multifunctional conductive hydrogel was fabricated via a one‐pot method using CMC, polyacrylamide, and tannic acid, cross‐linked within a dual‐network structure (Figure 3g).^[38]^ The incorporation of CMC significantly enhanced the mechanical properties by forming extensive hydrogen‐bonding networks and acting as sacrificial bonds for efficient energy dissipation. These structural contributions resulted in exceptional stretchability (up to 747% strain), high toughness, and a linear sensing response (gauge factor [GF] = 2.52) across a wide strain range (Figure 3h, i). Furthermore, the hydrogel exhibited notable anti‐freezing performance (down to −34.39°C), stable water retention over 30%–90% relative humidity, and high ionic conductivity, underscoring its suitability for durable, flexible strain sensors capable of operating under harsh environmental conditions.

### 3.2 Supramolecular network strategies: Physical interactions and tunable configurations

Supramolecular network design represents a pivotal strategy for enhancing the mechanical properties of cellulose gels by programming their physical architecture. Key design methodologies include constructing dual or multi‐network architectures, incorporating Dynamic covalent bonds (DCBs), enabling self‐healing capabilities, and introducing stimuli‐responsive motifs. These approaches collectively enhance the mechanical performance by enabling efficient energy dissipation through reversible bond rupture and reformation, distributing stress homogeneously, and maintaining structural integrity under deformation. Consequently, such tailored network designs lead to simultaneous improvements in strength, toughness, and fatigue resistance, effectively bridging molecular‐level functionalization and macroscale structural alignment in the hierarchical design of advanced cellulose‐based materials.

*Multi‐network design*: Double‐network (DN) and multi‐network (MN) gels overcome the limitations of single‐network systems by integrating complementary polymer networks—typically a rigid, brittle network with a soft, ductile network. Network structures of DN/MN gels are formed through chemical crosslinking relying on covalent bonds which are permanent and dissipative to external force,^[40]^ or physical crosslinking which are dynamic and restorative and utilizes reversible non‐covalent interactions such as hydrogen bonds,^[41]^ van der Waals forces,^[42]^ hydrophobic interactions,^[43]^ crystalline domains,^[44]^ electrostatic interactions^[45]^ and chain entanglements.^[46]^ Additionally, host‐guest interactions​ serve as a highly designable and specific type of physical crosslinking, leveraging molecular recognition between complementary host and guest molecules to form reversible and stimuli‐responsive supramolecular networks, such as cyclodextrin‐adamantane^[47]^ and crown ether‐ammonium.^[48]^ This unique mechanism, distinguished by its precise molecular specificity, provides a means to engineer gels with highly predictable and tunable mechanical strength, toughness, and resilience by allowing for precise control over crosslink density and binding affinity at the molecular level.^[49]^ Hybrid crosslinking containing both crosslinking methods can fully utilize both advantages.[^50^, ^51^, ^52^] The enhanced mechanics stem from efficient energy dissipation: the brittle network fractures sacrificially under stress, while the ductile network redistributes load to maintain integrity.^[53]^

**FIGURE 4:**
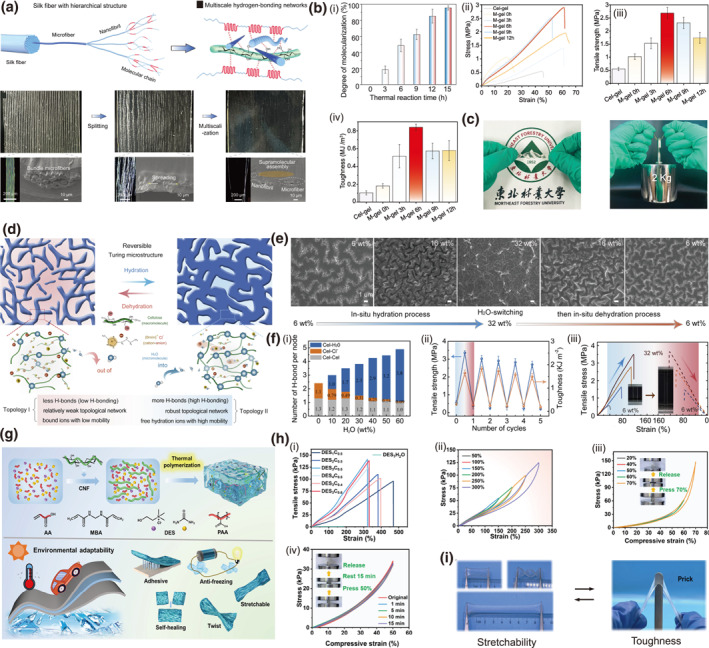
Double network design of functional cellulose gels. (a–c) Triple network cellulose ionogel (M‐gel): (a) Schematic illustration and optical microscopy of the triple network design. (b) Mechanical properties and reinforcement mechanism of M‐gel: (i) Degree of molecularization of silk fibers in M‐gel, (ii) Tensile stress–strain curves, (iii) Tensile strength and (iv) Toughness of M‐gel under different reaction times. (c) Optical images showing the flexibility and robustness of M‐gel. Reproduced with permission.^[55]^ Copyright 2023, Wiley‐VCH. (d–f) Topological network cellulose gel (Cel‐IL): (d) Schematic illustration of the reversible topological network. (e) Continuous SEM images of Cel‐IL with different H_2_O wt% during the in situ hydration‐dehydration process. (f) Mechanical properties and reinforcement mechanism of Cel‐IL: (i) Molecular dynamics simulation of number of H bonds per node. (ii) Tunable mechanical properties of the Cel‐IL in successive hydration‐dehydration cycles. (iii) Tensile stress‐strain curves in one cycle of hydration transition between two topological states. Reproduced with permission.^[56]^ Copyright 2020, Elsevier. (g–i) Dynamic H‐bonded network ionogel (DESC): (g) Schematic of the fabrication of all‐round DESC and flexible solid‐state supercapacitors. (h) Mechanical properties of DESC: (i) Tensile curves of DESC under different deep eutectic solvent (DES) and CNF ratios (ii) Cyclic tensile curves of DES_3_C_0.5_ gel under different strains. (iii) Compressive loading‐unloading curves and (iv) Compressive stress–strain curves for DES_3_C_0.5_. Reproduced with permission.^[57]^ Copyright 2025, Wiley‐VCH.

Multiscale network design, integrating structural elements across micro‐to‐nanoscopic scales, effectively disperses stress and inhibits crack propagation through synergistic interactions, thereby simultaneously enhancing strength and toughness.^[54]^ This approach overcomes the conventional trade‐off between rigidity and fracture resistance in soft materials. As an example, a bioinspired strategy employs in situ disassembly of silk fibers within a cellulose matrix under ionothermal stimulation, constructing a triple‐network architecture comprising microfibers, nanofibrils, and molecularized fibroin chains interwoven by multiscale H‐bonding (Figure 4a).^[55]^ The degree of molecularization of silk fibers increases with thermal reaction time, indicating enhanced nanofibrillation and formation of a denser hydrogen‐bonded network, where the increased surface area and interaction sites between cellulose and fibroin chains optimize the supramolecular topology. This design enables the ionogel to achieve an elastic modulus of 31.5 MPa and a toughness of 1540 kJ·m^−3^, capable of lifting weights over 8000 times its own mass (Figure 4b,c). Additionally, the ionogel exhibits high ionic conductivity (49.6 mS·cm^−1^), highlighting its potential for flexible electronics.

In dual‐network hydrogel designs, hydrogen‐bond (H‐bond) networks serve as dynamic crosslinks that enable intelligent mechanical regulation through reversible bonding and dissociation. This allows the material to adapt its structural topology, significantly enhancing toughness and strength in response to environmental stimuli like moisture content. By varying the water content, a representative dynamic cellulose hydrogel undergoes reversible switching of its hydrogen‐bond topological network between a loose adhesive state (Topology I) and a dense robust network (Topology II) (Figure 4d).^[56]^ This transition, driven by H‐bond reorganization beyond a critical water threshold (Figure 4e), yields remarkable mechanical enhancement: toughness surges from 246.2 to 1652.9 kJ·m^−3^ and tensile strength boosts from 0.30 to 3.50 MPa (Figure 4f).

Additionally, the gel demonstrates superior self‐healing and ionic conductivity, highlighting its potential for adaptive electronics and flexible robotics. As a further example, a sustainable ion‐gel electrolyte composed of polyacrylic acid (PAA), deep eutectic solvent (DES), and cellulose nanofibers (CNF) forms a double‐network structure through dynamic H‐bond crosslinking (Figure 4g).^[57]^ This architecture delivers enhanced mechanical properties with a tensile strength of 142 kPa, strain of 322%, and toughness of 203 kJ·m^−3^ (Figure 4h,i). The gel also exhibits high ionic conductivity, self‐healing capability, and wide‐temperature adaptability, demonstrating promise for flexible supercapacitors.

*Dynamic covalent bonds network design*: DCBs are a unique class of chemical bonds that combine the strength of traditional covalent bonds with the ability to reversibly break and re‐form under specific physiological or external stimuli. Common types of DCBs utilized in advanced hydrogel design include imine bonds (Schiff base),^[58]^ borate ester bonds,^[59]^ disulfide bonds,^[60]^ diselenide bonds^[61]^ and those formed via Diels‐Alder reactions.^[62]^ The principal advantage of incorporating these dynamic bonds into gel networks for mechanical regulation lies in their ability to confer tunable viscoelasticity and self‐healing capabilities. The reversible breakage and reformation of these bonds under stress allow for efficient energy dissipation, significantly enhancing the material's toughness and enabling precise control over key mechanical properties such as storage modulus and stress relaxation rates by simply adjusting the bond density or the ratio of interacting components. This dynamic nature facilitates the creation of adaptable hydrogel systems that can better mimic the time‐dependent mechanical properties of natural biological tissues.

**FIGURE 5:**
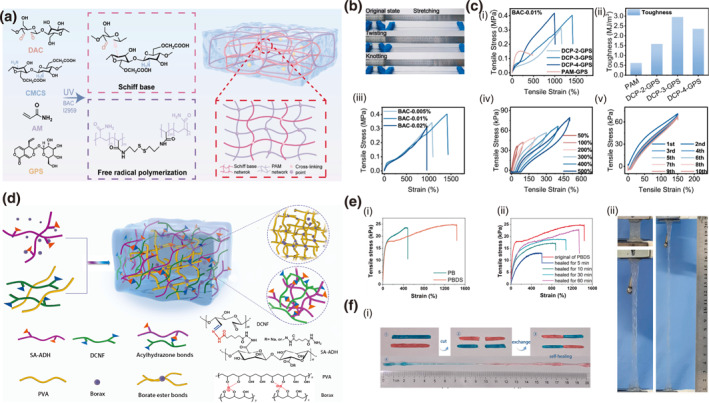
Dynamic covalent network design of cellulose gels. (a–c) Schiff base cellulose gel (DCP‐GPS): (a) Schematic illustration of the synthesis of DCP‐GPS. (b) Optical images showing twisting, stretching, and knotting of DCP‐GPS. (c) Mechanical properties of DCP‐GPS: (i) Tensile stress‐strain curves and (ii) Toughness of DCP‐GPS with different CMCS concentrations. (iii) Tensile stress‐strain curves with different crosslinker (BAC) concentrations. (iv) Loading‐unloading curves and (v) Multicycle tensile stress‐strain curves of DCP‐3‐GPS. Reproduced with permission.^[64]^ Copyright 2025, Elsevier. (d–f) Double dynamic covalent bonding network cellulose gel (PBDS). (d) Schematic illustration of PBDS fabrication. (e) Mechanical properties of PBDS: (i) Stress‐strain curves of PB and PBDS. (ii) Stress‐strain curves of PBDS under different self‐healing duration. (f) Optical images of PBDS: (i) Self‐healing process of PBDS. (ii) Stretchability of PBDS hydrogel. Reproduced with permission.^[65]^ Copyright 2023, Elsevier.

The Schiff‐base reaction represents a particularly versatile strategy for constructing DCBs in hydrogel systems, primarily forming imine bonds (C=N) through the condensation between aldehyde groups and primary amines. A key advantage of imine bonds is their relatively rapid reversible kinetics under physiological conditions, especially in response to mild acidic stimuli, which is common in specific biological microenvironments like tumor sites or wound areas.^[63]^ A double‐network hydrogel (DCP) utilizing dynamic Schiff base bonds demonstrates remarkable self‐healing and energy‐dissipation capabilities, with its GPS‐loaded variant (DCP‐GPS) serving as an effective drug delivery platform for wound therapy.^[64]^ Formed between dialdehyde cellulose (DAC) and carboxymethyl chitosan (CMCS), the reversible imine network provides DCP‐GPS with a dynamically adaptive structure (Figure 5a), achieving exceptional mechanical strength (402.37 kPa), ultrahigh stretchability (>1400% strain), and outstanding toughness (2958.44 kJ m^−3^) (Figure 5b,c).

Borate ester bonds, formed between diols and borate ions, exhibit rapid reversibility under mild conditions due to dynamic B–O crosslinking.^[66]^ Similarly, acylhydrazone bonds generated from aldehyde and hydrazide groups offer tunable kinetics and enhanced stability.^[67]^ The synergistic combination of these DCBs enables efficient energy dissipation through controlled rupture and reformation under stress, significantly enhancing material toughness and autonomous self‐healing. As a specific example, a dual‐network hydrogel (PBDS) incorporates borate ester bonds from PVA and borax, alongside acylhydrazone bonds formed between aldehyde nanocellulose (DCNF) and adipic acid dihydrazide‐modified alginate (SA‐ADH) (Figure 5d).^[65]^ This design achieves exceptional mechanical performance, including ultrahigh stretchability (1440% strain) and rapid self‐healing (92.7% efficiency in 60 min at ambient temperature) (Figure 5e,f). The hydrogel also demonstrates ionic conductivity and biocompatibility, facilitating its use in strain sensing and biomedical devices.

Stimuli reinforced network construction: Stimulus‐induced strengthening is an effective strategy where external triggers such as solvent, temperature and pH, dynamically reconfigure a material's supramolecular architecture. This enhancement is initiated as the stimulus disrupts weaker transient bonds and promotes the formation of stronger bonds, leading to structural transformations such as chain coiling and nanodomain formation.^[68]^ This mechanistic framework is broadly applicable to smart hydrogel systems, where stimuli‐responsive reorganization underpins enhanced functional performance. UV light reduces Fe^3+^ to Fe^2+^, breaking the weak coordination bonds and exposing controllable reaction sites to spatially direct polymerization in hydrogels, enabling applications such as smart circuits and information encryption.^[69]^

**FIGURE 6:**
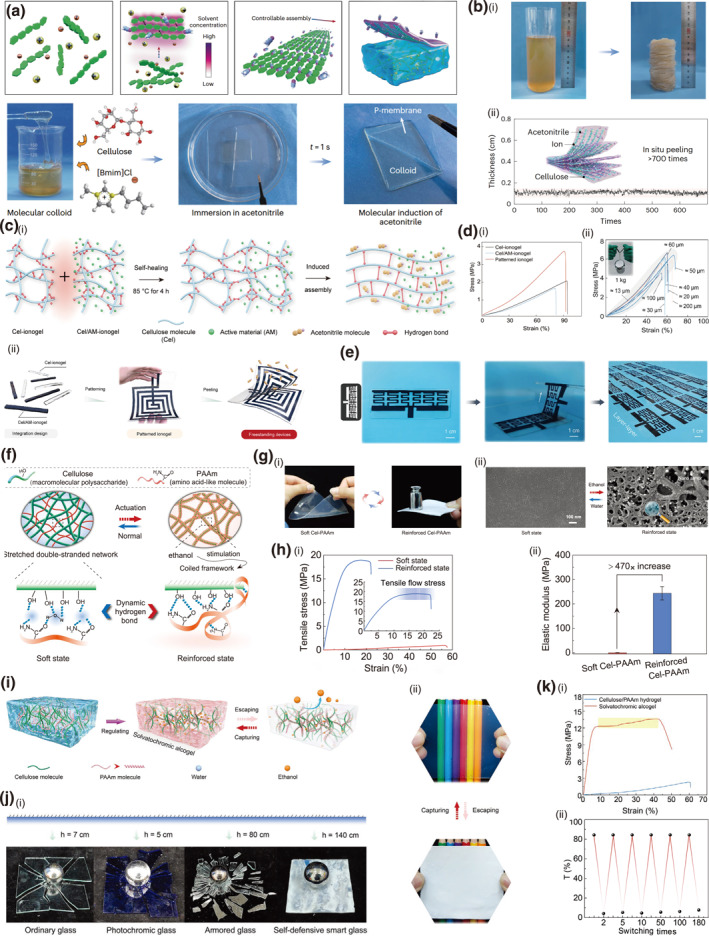
Stimuli reinforced network design of cellulose gels. (a, b) Solvent‐induced self‐assembly peeled cellulose ionogel membrane (P‐membrane). (a) Schematic illustration and photographs of preparation of P‐membranes. (b) Peeling properties of P‐membranes: (i) Photographs showing cellulose/[Bmim]Cl colloid and its derived P‐membranes (>700 pieces). (ii) Thickness distribution of the P‐membranes. Reproduced with permission.^[70]^ Copyright 2023, Wiley‐VCH. (c–e) Ultrathin solvent‐induced peeled and patterned cellulose/Active Materials ionogel (patterned Cel/AM‐ionogel). (c) (i) Schematic illustration and (ii) Photographs of preparation of patterned Cel/AM‐ionogels. (d) Mechanical properties of patterned Cel/AM‐ionogels. (i) Comparison of the mechanical tensile performance between the Cel‐ionogel, the Cel/AM‐ionogel, and the patterned ionogel. (ii) Tensile stress‐strain curves for peeled patterned Cel‐AM ionogel with varying thicknesses. (e) Optical images depicting the in situ exfoliation process for the fabrication of freestanding devices with customizable patterns. Reproduced with permission.^[16]^ Copyright 2023, Wiley‐VCH. (f–h) Ethanol induced switchable cellulose/PAAm hydrogel (Cel‐PAAm). (f) Schematic illustration of mechanics of switchable Cel–PAAm. (g) (i) Photos and (ii) SEM images of Cel–PAAm in its different states. (h) Mechanical properties of Cel‐PAAm. (i) Tensile stress–strain curves of Cel–PAAm in its soft and reinforced states. (ii) Elastic modulus of Cel–PAAm in its two states. Reproduced with permission.^[71]^ Copyright 2022, Wiley‐VCH. (i–k) Ethanol stimulated solvatochromic Cel/PAAm alcogel (Sol‐gel). (i) Schematic of the fabrication of Sol‐gel. (j) Photos of Sol‐gel: (i) Free‐fall impact resistance testing of different materials with ion ball of 256 g. (ii) Digital images of Sol‐gel showing switchability in light modulation behavior via ethanol escape–capture operations. (k) Properties of Sol‐gel: (i) Tensile stress and strain curves before and after ethanol stimulation. (ii) Cyclic switchability of the light‐regulated behavior of alcogel. Reproduced with permission.^[72]^ Copyright 2023, Wiley‐VCH.

Based on the milk‐skin formation mechanism, a universal solvent‐induced self‐assembly strategy is developed for fabricating biomacromolecular ionogel membranes.^[70]^ This is achieved by immersing Cellulose/PAAm/[Bmim]Cl ionogel into acetonitrile, where acetonitrile molecules disrupt the original cellulose‐ionic liquid hydrogen bonds and form stronger preferential hydrogen bonds with cellulose via C≡N groups, thereby guiding the orderly supramolecular self‐assembly and enabling facile exfoliation (Figure 6a). The resulting membranes exhibit excellent peelability, enduring over 700 cycles while maintaining uniform thickness (Figure 6b). Furthermore, they possess high ionic conductivity (up to 14.1 mS cm^−1^), good stretchability (>130%), and tunable mechanical strength, highlighting their potential for scalable production in flexible electronics and sensors. Building upon this platform, subsequent research has further developed its application potential by incorporating self‐healing to fabricate ultrathin freestanding devices.^[16]^ Upon heat treatment, a pure cellulose ionogel (Cel‐ionogel) and an active material‐loaded ionogel (Cel/AM‐ionogel) can be healed into a seamlessly patterned structure (Figure 6c). This patterned Cel/AM‐ionogel possess a substantial enhancement in mechanical performance, exhibiting a tensile strength of 3.74 MPa and an elastic modulus of 5.47 MPa, thus surpassing the properties of its individual components (Figure 6d). Along with the preceding exfoliation/peeling strategy using acetonitrile, the patterned Cel/AM‐ionogel can be fabricated into an ultrathin freestanding device, demonstrating strong potential for applications such as e‐skins, neural networks, and thermoelectric devices (Figure 6e).

A biomimetic strategy inspired by sea cucumber dermis achieves dramatic mechanical enhancement in cellulose/polyacrylamide hydrogels via ethanol‐induced supramolecular reconfiguration.^[71]^ Ethanol stimulation triggers a reversible transition where PAAm chains coil, disrupting the original hydrogen‐bonding network and forming dense coordination and hydrogen bonds with cellulose (Figure 6f,g). This structural shift from a loose double‐network to a tightly coiled configuration yields a 21‐fold higher tensile strength (18.39 MPa), a 470‐fold increase in elastic modulus (reaching 243.6 MPa), and a 3000‐fold improvement in scratch resistance (Figure 6h). This Cel‐PAAm hydrogel can be further engineered into a self‐defensive smart window alcogel exhibiting two distinct states governed by ethanol escape and capture (Figure 6i).^[72]^ In its opaque state after ethanol escape, the alcogel demonstrates exceptional impact resistance, with the fabricated smart window sustaining 42.8 kJ m^−2^, surpassing ordinary glass by tenfold and even exceeding armored glass. Conversely, the ethanol‐captured state offers high transparency up to 85%, with a rapid switching cycle of less than 8 s (Figure 6j,k). This combination of rapid optical modulation and superior mechanical strength holds significant promise for developing energy‐efficient, impact‐resistant smart windows in buildings and safety protection systems.

### 3.3 Macroscale structure strategies: Structural alignment and hierarchical design

At the macroscale structure level, the design of gels shifts its focus towards the spatial arrangement of cellulose building blocks and the architecture of macroscopic pores. This approach aims to construct biomimetic hierarchically ordered structures that fundamentally enhance mechanical performance by controlling the larger‐scale organization of the material's framework. This approach primarily involves structural orientation via external fields and the creation of aligned porous structures by freeze‐casting, while advanced techniques like 3D printing^[73]^ and electrospinning^[74]^ also serve as potential tools. This oriented arrangement or oriented pore structure has been proven to enhance material performance.^[75]^ The reinforcement mechanism stems from the fact that these designed, ordered, or hierarchically porous structures guide stress to transfer efficiently along specific pathways, such as the direction of fiber alignment or pore walls, which significantly reduces stress concentration points common in random networks. This directed stress transfer and optimized energy dissipation pathway work synergistically with the higher packing density and stronger interactions within the aligned structures, leading to a macroscopic synergy in the improvement of modulus, strength, and toughness.

*Structural orientation design via external fields*: Structural orientation induced by external fields such as shear flow,^[76]^ gravity, mechanical stretching,^[77]^ and electric or magnetic fields,^[78]^ serves as a fundamental strategy for enhancing the mechanical properties of cellulose gels. The principle involves these fields applying directional forces that overcome the random thermal motion of polymer chains, prompting their alignment along a specific axis. This ordered, anisotropic architecture significantly strengthens the gel by enabling more efficient stress transfer along the aligned chains, minimizing stress concentration, and providing optimized pathways for energy dissipation. Consequently, this leads to remarkable improvements in modulus, tensile strength, and toughness in the orientation direction.

**FIGURE 7:**
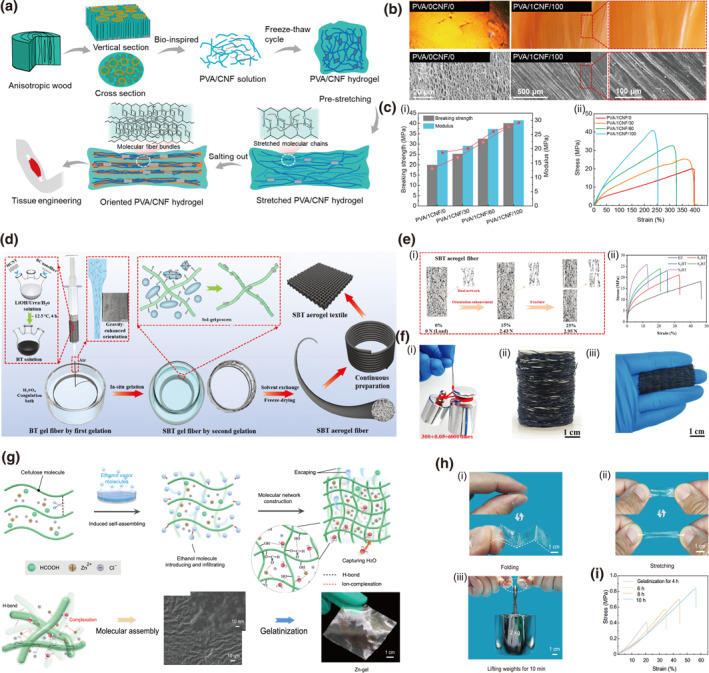
Structural orientation via external fields of cellulose gels. (a–c) Salting‐assisted pre‐stretching treated PVA/CNF hydrogel. (a) Schematic illustration of fabrication of PVA/CNF hydrogel. (b) Optical micrograph and SEM images of the original PVA hydrogel and anisotropic PVA/1CNF/100. (c) Mechanical properties of PVA/CNF hydrogel: (i) Breaking strength and modulus and (ii) Stress‐strain curves of PVA/1CNF/y hydrogels with different pre‐stretched deformation variables. Reproduced with permission.^[80]^ Copyright 2023, American Chemical Society. (d–f) Gravity‐enhanced orientation of cellulose airgel fiber (SBT): (d) Schematic illustration of continuous preparation of SBT airgel fiber. (e) Mechanical properties and reinforcement mechanics of SBT airgel fiber: (i) Mechanism showing the highly oriented alignment of cellulose chains. (ii) Tensile stress‐strain curves of SBT airgel fibers at different silane concentrations. (f) Optical images showing (i) strength, (ii) continuous preparation capability and (iii) flexibility of SBT airgel fibers. Reproduced with permission.^[81]^ Copyright 2025, Wiley‐VCH. (g–i) Ethanol vapor‐induced assembly of cellulose hydrogel (Zn‐gel). (g) Schematic illustration of Zn‐gel fabrication. (h) Optical images showing (i) foldability, (ii) flexibility and (iii) strength of the Zn‐gel. (i) Tensile stress–strain curves of the Zn‐gels during different ethanol‐induction times. Reproduced with permission.^[82]^ Copyright 2024, Wiley‐VCH.

Mechanical prestretching serves as a pivotal processing technique that fundamentally transforms the inherently disordered state of molecular chains within gel networks. It induces chain orientation along the applied stress direction, thereby facilitating the formation of highly ordered nanofibrous architectures and promoting crystallinity through the disruption of weak non‐covalent bonds and enhanced molecular packing.^[79]^ Illustrating this principle, a CNF/PVA hydrogel features a wood‐inspired, highly oriented arrangement of molecular chains, achieved through an integrated salting‐assisted prestretching treatment (Figure 7a).^[80]^ Prestretching induces chain alignment, fixed by salt‐induced aggregation into robust fiber bundles (Figure 7b). This meticulously engineered anisotropic structure endows the hydrogel with exceptional mechanical properties, including an ultrahigh breaking strength exceeding 40 MPa, a strain approaching 250%, and a remarkable toughness of over 60 MJ·m^−3^ (Figure 7c). It also exhibits excellent tear resistance and biocompatibility, showcasing promise for applications like artificial tendons and ligaments.

Gravity‐induced orientation serves as a specific form of mechanical stretching specifically designed for sol‐state gel systems, utilizing the material's own weight as a naturally uniform, axial tensile force. This method represents a unique case where the stretching force is intrinsic and evenly distributed, contrasting with externally applied and often localized mechanical forces. A pertinent illustration can be seen in the super‐strong bacterial cellulose (BC)/hydroxylated carbon nanotube (HCNT) airgel fibers fabricated through a dual gelation strategy under gravity‐enhanced orientation.^[81]^ This process aligns BC molecular chains and HCNTs under gravitational force during dry‐wet spinning, followed by an in situ silanization that forms a chemically interlocked dual network (Figure 7d,e). This structurally refined architecture yielded a remarkable tensile strength of up to 26.0 MPa and a modulus of 129.8 MPa for the final fiber, which can be fabricated into a flexible airgel textile (Figure 7f).

Vapor‐induced phase separation (VIPS) enables precise control over molecular self‐assembly and the formation of well‐defined porous architectures through a gradual solvent‐exchange process, where the permeation of precipitant vapor modulates solution properties and initiates liquid‐liquid phase separation to guide the organized arrangement of polymer chains. The non‐contact nature of this method promotes uniform structural development by preventing localized rapid precipitation, as the slow diffusion of vapor molecules across the vapor‐liquid interface creates a dynamic equilibrium, allowing molecules to spontaneously reorganize into thermodynamically stable ordered structures. A seminal example of this principle is offered by a cellulose zinc‐ion gel electrolyte synthesized via an ethanol vapor‐induced assembly strategy, which represents a distinct physical stimulation method (Figure 7g).^[82]^ Ethanol vapor molecules infiltrate a cellulose DES system, thus triggering a precise hydrogen‐bond‐guided molecular self‐assembly into a densely interconnected network (Figure e). This uniquely structured gel achieved a tensile strength of 0.88 MPa while also exhibiting an outstanding ionic conductivity of 8.39 mS cm^−1^ (Figure 7h,i), demonstrating a successful combination of mechanical and ionic transport properties.

*Porous structure design by directional freeze‐casting*: Directional freeze‐casting, or ice‐templating, is a versatile method for fabricating gels with tailored porous architectures. The process involves freezing a gel precursor solution, during which growing ice crystals act as a template to push the solidifying gel matrix into an interconnected, often aligned, porous network. Subsequent sublimation of the ice under vacuum preserves this microstructure. The resulting porous structure significantly enhances the mechanical properties of the gel. The aligned and interconnected pores can act to effectively dissipate stress and deflect crack propagation, thereby improving the material's toughness and damage tolerance. This unique combination of controlled porosity and mechanical reinforcement makes freeze‐cast gels highly valuable for applications requiring lightweight yet robust materials, such as in tissue engineering scaffolds.

**FIGURE 8:**
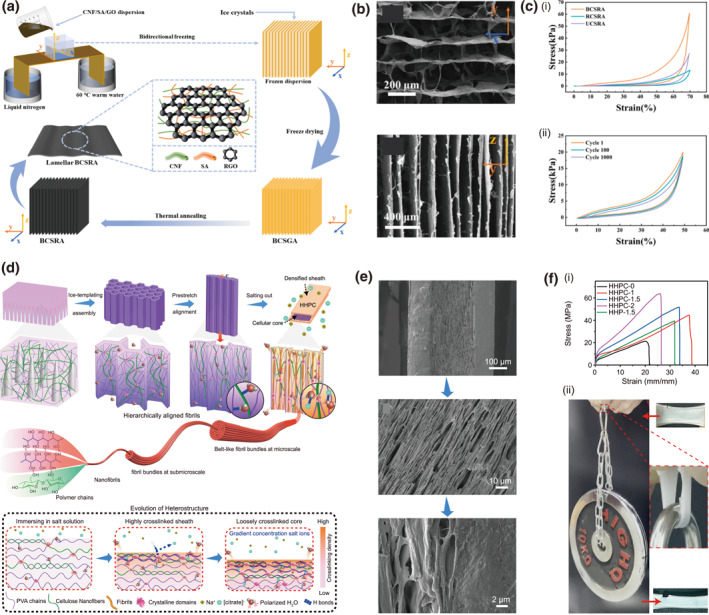
Directional freeze‐casting of cellulose gels. (a–c) Bidirectional freeze‐drying cellulose airgel (BCSRA): (a) Schematic illustration of preparation process for BCSRA. (b) SEM images showing the aligned porous structure of BSCRA. (c) Mechanical properties of BCSRA hydrogels: (i) Stress‐strain curves of aerogels under different freeze‐casting methods. (ii) Stress‐strain curves of BCSRA at 50% strain for 1000 cycles. Reproduced with permission.^[83]^ Copyright 2025, Elsevier. (d–f) Bidirectional freeze‐drying PVA/CNF hydrogel (HHPC). (d) Schematic illustration of design and fabrication of. (e) SEM images of HHPC−1.5 hydrogel. (f) Mechanical properties of HHPC hydrogels: (i) Tensile stress‐strain curves of HHPC hydrogels. (ii) Photograph showing mechanical strength of a HHPC‐1.5 hydrogel. Reproduced with permission.^[84]^ Copyright 2025, Springer Nature. BCSRA, bidirectional freeze‐drying cellulose.

Under a bidirectional freeze‐drying strategy, cellulose nanofibers/sodium alginate/reduced graphene oxide airgel (BCSRA) is fabricated. The BCSRA airgel features a highly ordered lamellar porous structure through precise control of ice crystal growth along dual temperature gradients (Figure 8a,b).^[83]^ This multi‐scale aligned architecture endows the material with exceptional mechanical properties, achieving an ultralow density of 7.21 mg·cm^−3^ and a compressive strength of 64.55 kPa at 70% strain, which is significantly higher than that of RCSRA (randomly frozen) with 13.35 kPa and UCSRA (unidirectional frozen) with 27.65 kPa (Figure 8c). The aligned pores also facilitate efficient ion transport, contributing to a high‐pressure sensitivity of 5.71 kPa^−1^ for sensing applications, while maintaining robust fatigue resistance (82.17% stress retention after 100 cycles at 70% strain). In a similar method, a hierarchically heterogeneous PVA/CNF hydrogel (HHPC) with core‐sheath architecture is fabricated comprising an aligned porous core and a compact sheath, which enables multi‐scale fibril orientation (Figure 8d,e).^[84]^ The hydrogel achieves ultrahigh mechanical properties, including toughness of 1031 MJ m^−3^, strength of 55.3 MPa, and fatigue resistance (Figure 8f). Additional functionalities like regeneration and adhesion are briefly noted, highlighting their potential for advanced applications.

## 4 ELECTRICAL REINFORCEMENT DESIGN OF FUNCTIONAL GEL

The inherent potential of cellulose gels as sustainable functional materials is often limited by their native electrical insulation properties, which restricts their application in modern electronic and energy devices.^[85]^ Electrical enforcement, therefore, becomes a critical strategy to transform these biopolymer networks into conductive or semi‐conductive platforms, enabling them to actively participate in electron or ion transport processes. Common modification approaches include the incorporation of conductive polymers, carbon nanomaterials, or ionic salts to create efficient charge‐transfer pathways within the gel matrix.[^24^, ^86^, ^87^, ^88^] Furthermore, structural engineering at the molecular level, such as constructing specific nanofluidic channels, can significantly enhance ion mobility and selectivity. These enhancements unlock the potential of cellulose gels for a wide range of advanced applications, including flexible sensors, energy storage devices like supercapacitors and batteries, and even innovative energy harvesters that convert ambient humidity into electricity.

### 4.1 Chemical modification and functional groups regulation

Chemical modification represents a fundamental strategy for tailoring the charge transport characteristics of cellulose‐based materials through the deliberate introduction of specific functional groups. Key modifying groups such as sulfonic acid (‐SO_3_H),^[89]^ carboxyl (‐COOH),^[90]^ phosphate (‐PO_3_H_2_),^[91]^ and quaternary ammonium (‐NR_3_^+^)^[92]^ serve as ion‐hopping sites or effective dopants, significantly enhancing ion migration efficiency or promoting the establishment of electron transport networks within the material. This molecular‐level design enables the modified cellulose to acquire tunable electrical properties, including markedly improved ionic conductivity and charge carrier mobility, while preserving its inherent renewable and biodegradable attributes. Such electro‐active cellulose‐based systems, which combine notable electrical performance with environmental friendliness, open promising avenues for the development of next‐generation green electronics and sustainable energy storage applications.

**FIGURE 9:**
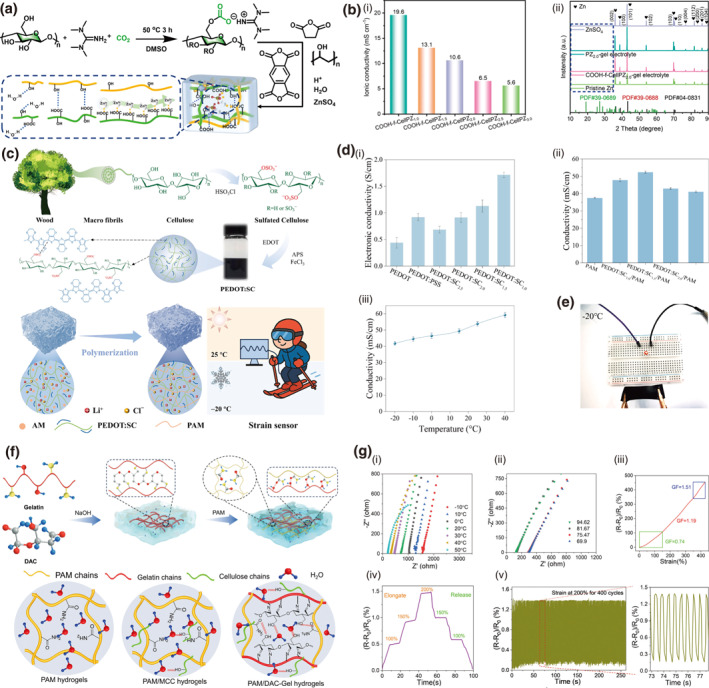
Chemical modification for tunable electrical properties in cellulose gels. (a, b) Carboxyl modified cellulose hydrogel (COOH‐f‐CellPZ‐gel). (a) Schematic illustration of the preparation of COOH‐f‐CellPZ‐gel. (b) Electrical properties and mechanics of COOH‐f‐CellPZ‐gel: (i) Ionic conductivity of COOH‐f‐CellPZx‐gel electrolyte. (ii) XRD patterns of the Zn anode at the pristine state and deposited states after cycling (1 mA cm^−2^/1 mAh cm^−2^) for 50 h. Reproduced with permission.^[93]^ Copyright 2023, Elsevier. (c–e) Sulfate modified cellulose hydrogel (PEDOT:SC). (c) Schematic illustration of the preparation process of SC, PEDOT:SC, and motion‐sensing performance of PEDOT:SC. (d) Electrical properties and mechanics of PEDOT:SC: (i) Electrical conductivity of PEDOT:PSS and PEDOT:SC with varying SC contents. (ii) Electrical conductivity of PAM and PEDOT:SC_x_/PAM hydrogels. (iii) Conductivity stability of the PEDOT:SC_1.5_/PAM hydrogel after storage at 20°C for 30 days. (e) Photographs showing an LED illuminated by the PEDOT:SC_1.5_/PAM hydrogel at −20°C. Reproduced with permission.^[94]^ Copyright 2025, Elsevier. (f, g) Ring opening modified cellulose. (f) Schematic illustration of synthesis and preparation flowchart of the PAM/DAC‐2Gel hydrogel network, and hydrogen bonding interactions in hydrogels. (g) Electrical and sensing properties of PAM/DAC‐2Gel strain sensor: (i) Nyquist diagrams of PAM/DAC‐2Gel hydrogel at different temperatures and (ii) different humidities. (iii) Variations in relative resistance and gauge factor (GF) of the strain sensor across different tensile strains. (iv) Time profile of relative resistance changes with consecutively applied strains. (v) Relative resistance changes of the strain sensor under 400 loading–unloading cycles at 200% strain. Reproduced with permission.^[95]^ Copyright 2024, Wiley‐VCH.

Chemical functionalization of cellulose through sulfonation or carboxylation introduces anionic groups that electrostatically interact with cationic conductive materials, thereby enhancing mechanical robustness via improved interfacial adhesion and promoting charge transport through optimized percolation pathways and reduced energy barriers. For instance, a carboxyl‐modified cellulose hydrogel electrolyte (COOH‐f‐CellPZ‐gel) fabricated via succinic anhydride grafting is reported.^[93]^ The grafted ‐COOH groups construct ordered ion channels, which facilitate rapid Zn^2+^ transport and induce preferential Zn deposition on the (002) crystal plane (Figure 9a). This specific orientation prevents vertical dendrite growth, effectively suppressing dendrites. Consequently, the electrolyte exhibits an enhanced ionic conductivity of 10.6 mS cm^−1^, attributed to improved ion hopping and reduced interfacial resistance. The COOH‐f‐CellPZ‐gel enables exceptional cycling stability (>70,000 cycles with 91% capacity retention) in zinc‐ion hybrid capacitors (Figure 9b). It also possesses good mechanical robustness (220 kPa tensile strength) and flexibility, demonstrating potential for durable flexible energy storage devices.

Similarly, a sulfonation approach​ was employed to fabricate a sulfated cellulose (SC)‐stabilized PEDOT dispersion (PEDOT:SC) via molecular engineering^[94]^ Here, high‐degree sulfonation introduces abundant ‐SO_3_H groups to enhance charge transfer and construct ordered ion channels for rapid Zn^2+^ transport. The sulfonic acid groups are negatively charged, enabling strong electrostatic interactions with the positively charged PEDOT chains, effectively doping PEDOT and promoting charge transport (Figure 9c). The designed channels and PEDOT/SC synergistic network achieve high conductivity of 52.4 mS cm^−1^, maintaining 41.7 mS cm^−1^ even at −20°C (Figure 9d,e). The hydrogel also exhibits excellent mechanical flexibility (650% strain), adhesion, and cryo‐resistance, enabling stable performance in extreme environments for flexible sensing applications.

Hydrophobic modification of cellulose reduces free water content and increases bound water, which optimizes the ion migration environment by minimizing random scattering and effectively concentrating charge carriers, thereby significantly enhancing electrical conductivity. This mechanism is exemplified by carbonyl functionalization via cellulose oxidation to DAC, a key step in preparing the PAM/DAC‐2Gel hydrogel.^[95]^ The introduced C=O groups synergize with dual‐network crosslinking through Schiff‐base reactions, significantly improving structural stability and charge transport. Incorporation of Na^+^ ions increases charge carrier density and promotes ion‐hopping conduction, while the optimized microporous architecture provides ordered pathways for ion migration, minimizing resistance (Figure 9f). PAM/DAC‐2Gel is further configured as strain sensors which demonstrates a GF of 1.51 with a highly reversible resistance response over 400 stretching cycles, enabling reliable monitoring of human joint movements (Figure 9g).

### 4.2 Supramolecular network configuration for functional gels

Supramolecular network engineering provides an equally powerful platform for enhancing the electrical performance of cellulose gels by strategically structuring ion and electron transport pathways. The design of these dynamic networks, which has proven effective in improving mechanical properties, can be tailored to facilitate efficient charge migration through the formation of continuous conductive highways or optimized ion‐hopping sites. By precisely arranging functional groups and controlling pore architecture at high conductivity and structural integrity, this approach establishes supramolecular network‐level engineering as a fundamental strategy for developing advanced electro‐active cellulose materials.

**FIGURE 10:**
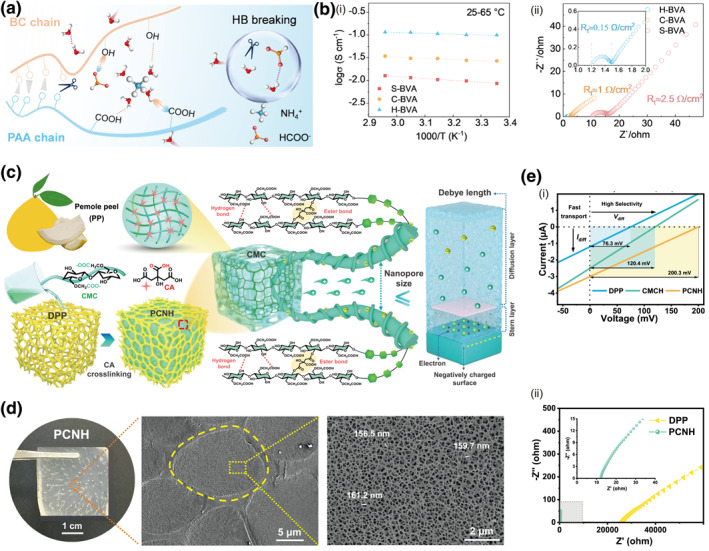
Supramolecular Network Design for tunable electrical properties in cellulose gels. (a, b) “Salting‐in” effect utilized bacterial cellulose‐reinforced hydrogel electrolyte (IBVA). (a) Schematic illustration of “Salting‐in” effect. (b) Electrical properties of IBVA hydrogels: (i) Ionic conductivities of various IBVA hydrogels under various temperatures. (ii) Electrochemical impedance spectroscopy curves of various IBVA hydrogel electrolytes. Reproduced with permission.^[96]^ Copyright 2025, American Chemical Society. (c–e) Debye screening effect enhanced delignified pomelo peel‐confined CMC nanofluidic hydrogel (PCNH). (c) Schematic illustration of the preparation process and nanopore structure of PCNH. (d) Photographic and Cryo‐SEM images of PCNH hydrogels. (e) Electrical properties of PCNH hydrogels: (i) I–V curves of DPP, CMCH, and PCNH. (ii) Nyquist plot of the electrochemical performances of PCNH and DPP. Reproduced with permission.^[96]^ Copyright 2025, Springer Nature. PCNH, peel‐confined CMC nanofluidic hydrogel.

The construction of ion channels in cellulose gels can be based on the idea of breaking the original dense hydrogel bonds. A novel BC‐reinforced hydrogel electrolyte (IBVA) masterfully balances high ionic conductivity and mechanical strength.^[96]^ Its core innovation lies in the strategic application of the “salting‐in” effect​ from chaotropic formate anions (HCOO^−^). These anions form weak hydrogen bonds with the PVA/PAA polymer chains, effectively loosening the dense network and creating expanded pathways for rapid ion transport, while a robust BC framework maintains structural integrity (Figure 10a). This molecular design yields an ultrahigh ionic conductivity of 105 ± 5 mS cm^−1^, alongside a tensile strength of 0.78 MPa (Figure 10b). When deployed in supercapacitors, it enables excellent electrochemical performance and stability, showing great promise for flexible energy storage devices.

Concurrently, another approach leveraging bio‐inspired frameworks confines carboxymethyl cellulose within a delignified pomelo peel skeleton, generating sub‐Debye‐length nanopores that induce electric double‐layer overlap and enhance cation selectivity via the Debye screening effect (Figure 10c,d).^[97]^ This configuration enables high open‐circuit voltage (1.32 V) and short‐circuit current density (693.2 μA cm^−2^), demonstrating efficient moist‐electric conversion (Figure 10e). Both strategies effectively balance structural integrity with enhanced electrical performance, providing sustainable pathways for advanced energy applications.

## 5 THERMAL REINFORCEMENT DESIGN OF GELS

The pursuit of thermal stability in cellulose gels is driven by their need to function reliably under extreme temperature fluctuations, where conventional hydrogels fail due to solvent evaporation or freezing.^[98]^ Strategic design focuses on two fronts: managing the solvent phase through cryoprotectants or non‐volatile ILs to suppress phase change,[^99^, ^100^] and reinforcing the polymer network via multi‐network architectures or dynamic bonds.[^101^, ^102^] These approaches operate on the principles of freezing‐point depression, vapor pressure reduction, and, crucially, dynamic energy dissipation, which collectively enable the creation of mechanically robust materials capable of withstanding prolonged thermal and mechanical stress for applications in harsh‐environment electronics and energy storage.

**FIGURE 11:**
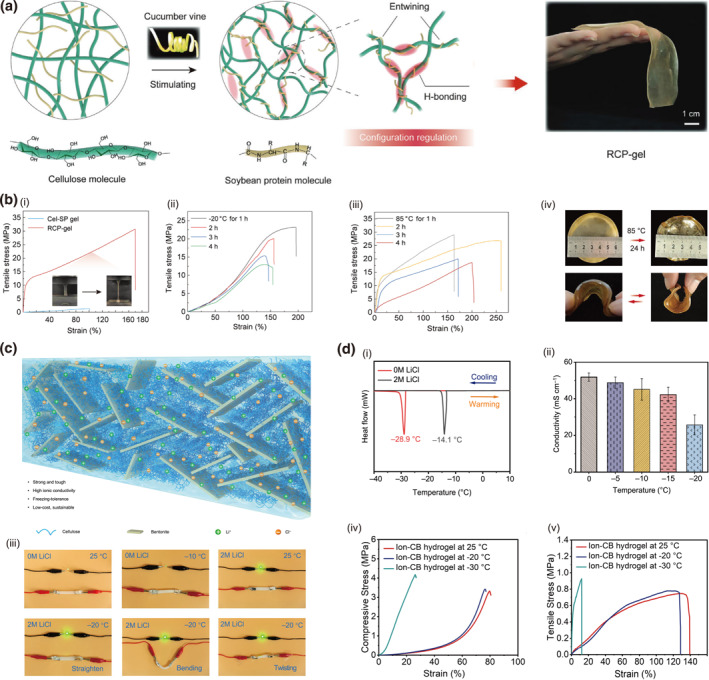
Thermal reinforced cellulose gels. (a, b) Acetone stimulated cellulose‐soybean protein ionogel (RCP‐gel). (a) Supramolecular configuration reinforcement strategy for developing robust RCP–gel. (b) Thermal and mechanical properties of RCP‐gel: (i) Stress–strain curves of Cel–SP gel and RCP–gel. (ii) Tensile stress–strain curves of RCP–gel when exposed to −20°C and (iii) 85°C. (iv) Optical images of the RCP–gel after exposure at 85°C for 24 h. Reproduced with permission.^[103]^ Copyright 2024, Wiley‐VCH. (c, d) LiCl reinforced anti‐freezing cellulose hydrogels (Ion‐CB): (c) Schematic of the proposed microstructure for cellulose/BT hydrogels. (d) Anti‐freezing performance of Ion‐CB hydrogels: (i) DSC curves of cellulose/BT hydrogel and Ion‐CB hydrogel. (ii) Ionic conductivity of Ion‐CB hydrogels with varying temperatures. (iii) Photos of luminance of LEDs using cellulose/BT hydrogel and Ion‐CB hydrogel as the conductor in the flat, bending, and twisting state at varying temperatures.

Reinforcing the polymer network via multi‐network architectures or dynamic bonds has been established as a viable strategy for enhancing the thermal resilience of cellulose‐based materials. Acetone stimulation triggers structural reorganization in cellulose‐soybean protein ionogels, transforming an extended double‐network into an entangled configuration via selective solvent exchange.^[103]^ This process promotes denser hydrogen bonding among hydroxyl and carboxyl groups, effectively suppressing chain relaxation under thermal stress (Figure 11a). The material achieves tensile strengths exceeding 15 MPa above 85°C and over 10 MPa at −20°C, with the storage modulus dominating from −80°C to 150°C (Figure 11b).

Modification of the ionic environment represents another effective route for cryogenic tolerance, primarily by depressing the freezing point of water to maintain ion transport at subzero temperatures. Incorporating bentonite nanoplatelets and LiCl salts into cellulose hydrogels synergistically enhances cryogenic tolerance through coordinated Al–O–C bonds and freezing‐point depression.^[104]^ The LiCl significantly lowers the aqueous phase freezing point to −28.9°C while maintaining efficient ion transport (Figure 11c). The hydrogel retains an ionic conductivity of 25.8 mS·cm^−1^ at −20°C and mechanical stability during thermal cycling (Figure 11d). Together, these strategies illustrate how external stimuli and ionic engineering can overcome the conventional trade‐off between mechanical integrity and thermal performance, providing reliable operation of cellulose gels under extreme environmental conditions.

### 5.1 Applications of functional cellulose gels

The escalating scarcity of petroleum resources has amplified the urgency for developing sustainable alternatives. Cellulose, as the most abundant natural polymer, is recognized as a pivotal renewable resource owing to its wide availability, low cost, and versatile physicochemical properties. Moreover, cellulose gels not only possess intrinsic mechanical superiority but can also be strategically tailored to enhance mechanical strength, electrical properties, and resilience to extreme temperatures. These advantages make cellulose gels highly attractive for cutting‐edge fields like flexible robotics, electronics and human‐machine interaction.

### 5.2 Flexible actuators and robotics

Stimuli‐responsive actuation refers to the ability of a material or system to undergo deformation or motion in response to external stimuli. This triggered actuation closely mimics the contraction and relaxation of muscles and has brought inspiration into the development of biomimetic flexible robotics and actuators.^[105]^ Two primary design strategies are commonly employed in developing these systems: (i) constructing bilayer, multilayer, or gradient structures that utilize marked swelling differences between distinct materials or layers,[^106^, ^107^, ^108^, ^109^] and (ii) designing homogeneous or composite structures in respond to gradient stimuli.[^25^, ^110^, ^111^]

**FIGURE 12:**
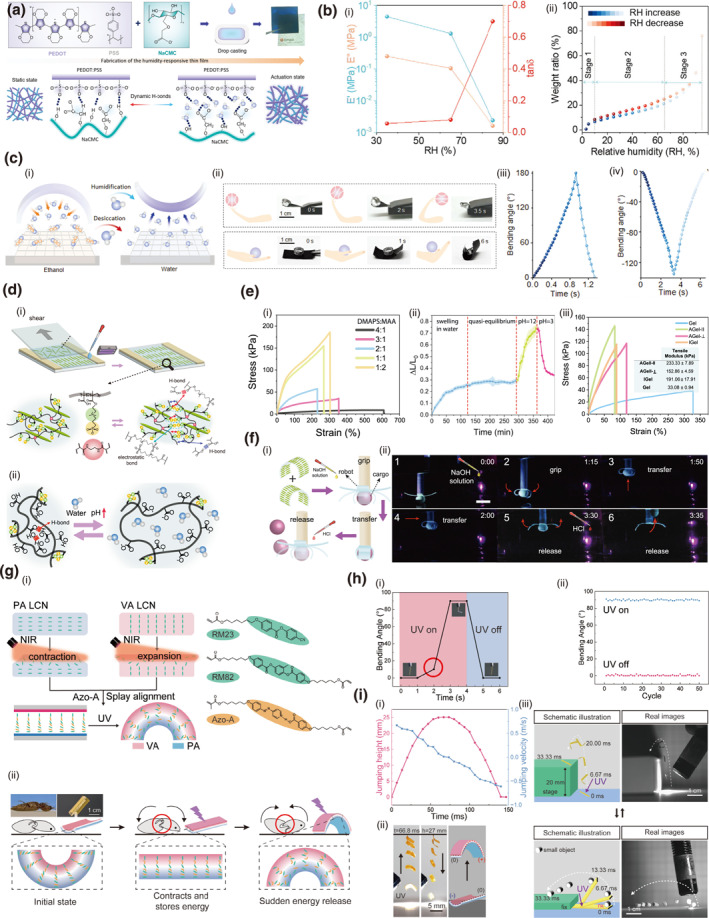
Flexible actuator and robotic applications of cellulose gels. (a–c) Humidity‐responsive hydrogel actuator‐based robot arm (PPC). (a) Schematic illustration of the fabrication of the PPC film. (b) Humidity‐responsive properties of PPC film. (i) DMA of the PPC films with humidity control. (ii) DVS analysis of the PPC films. (c) Humidity‐responsive behavior of PPC films: (i) Schematic diagram of the actuation mechanism of the PPC thin film. (ii) Proof‐of‐concept demonstrations of the practical applications of the athlete robot based on the PPC films. (iii) Time‐dependent variation of the bending angle of the PPC film stimulated by ethanol and (iv) water vapor. Reproduced with permission.^[113]^ Copyright 2024, Wiley‐VCH. (d–f) Shear‐induced anistropic pH‐response hydrogel actuator‐based gripper (GelWC). (d) Schematic illustration of (i) Fabrication of the anisotropic GelWC hydrogels and (ii) Swelling/deswelling mechanisms in response to pH. (e) Mechanical and pH‐responsive properties of GelWC hydrogels: (i) Stress‐strain curves of different GelWC hydrogels. (ii) Swelling behavior of GelWC hydrogels in water (pH ∼ 7) and in buffers with pH 12 and 3. (iii) Stress‐strain curves of GelWC hydrogels in parallel and perpendicular directions to CNC alignment. (f) (i) Schematic illustration and (ii) photos of the gripper application of anisotropic GelWC hydrogels. Reproduced with permission.^[114]^ Copyright 2023, Springer Nature. (g–i) NIR‐responsive hydrogel actuator based jumping robot (Azo‐LCN). (g) Schematic illustration of (i) Fabrication and (ii) Mechanism of bending behavior of Azo‐LCN hydrogels. (h) NIR‐responsive properties of Azo‐LCN hydrogels: (i) Bending angle of the film actuator. (ii) Cycling test results of Azo‐LCN actuators. (i) Jumping behavior of Azo‐LCN hydrogel‐based robot: (i) Height and speed during jump. (ii) Photos of jumping behavior. (iii) Photos of staircase climbing and stone throwing demonstration. Reproduced with permission.^[115]^ Copyright 2024, Wiley‐VCH. Azo‐LCN, azobenzene liquid crystal network.

Cellulose offers distinct advantages for humidity‐responsive actuators due to its nanoscale‐induced high specific surface area and abundant hydroxyl groups, which facilitate rapid and reversible moisture absorption through dynamic hydrogen bonding, thereby enabling efficient energy conversion from environmental humidity changes to mechanical deformation.^[112]^ A humidity‐responsive actuator was developed through supramolecular assembly of PEDOT:PSS and NaCMC (Figure 12a–c), forming a hydrogen‐bonded network that enables programmable humidity‐driven actuation.^[113]^ The composite film exhibits remarkable mechanical properties (12 MPa tensile strength, 400% fracture strain) and significant swelling capacity (76% mass change at 90% RH), facilitating complex shape‐morphing including twisting and folding deformations (Figure 12c). The actuator achieved practical robotic functions by lifting a cargo weighing 26 times its own mass within 3.5 s. The system further maintains excellent self‐healing properties, restoring 96% of mechanical performance through humidifying‐drying cycles.

The building of anisotropic structures to harness differential swelling for biomimetic motion emerges as a powerful approach to achieve programmable, directional deformation. A soft hydrogel composed of shear‐induced alignment of cellulose nanocrystals within a zwitterionic hydrogel matrix (Figure 12d–f), can be fabricated into a pH‐triggered gripper that lifts objects up to 26 times its own weight.^[114]^ The hydrogel matrix exhibited robust mechanical properties with a tensile strength of 0.78 MPa and a fracture strain of 3300% (Figure 12e). A magnetic patch by co‐precipitating Fe^2+^ and Fe^3+^ ions onto cellulose nanocrystals enables remote navigation and steering through confined fluid‐filled environments via external magnetic fields (Figure 12f).

Building upon fundamental actuation mechanisms, such functional materials can be further engineered to achieve sophisticated, life‐like motions beyond simple shape morphing, thereby advancing the development of flexible robots capable of complex locomotion. A soft jumping robot composed of a Janus azobenzene liquid crystal network (Azo‐LCN) film with splay alignment (Figure 12g–i), exhibits remarkable light‐driven continuous jumping capabilities with jumping heights of 35 body lengths and takeoff speeds of 670 BL s^−1^.^[115]^ The film demonstrates robust mechanical properties with a fracture stress of 36.27 MPa and strain of 6.5%, while achieving rapid photo‐responsive deformation through *trans*‐cis photoisomerization that triggers elastic energy release within 66.8 ms (Figure 12h). The robot successfully demonstrated practical applications including climbing a 20‐mm height staircase and throwing objects through precisely controlled jumping motions (Figure 12i).

### 5.3 E‐skins

As a frontier technology for health monitoring, e‐skins are designed to detect pressure, strain, and shear forces by converting mechanical deformation into electrical signals via piezoresistive, capacitive, or piezoelectric mechanisms. This requires high biocompatibility, mechanical flexibility, adhesion, excellent ionic conductivity, and multi‐stimuli responsiveness.[^116^, ^117^] This makes cellulose gels, which closely mimic natural human skin and are derived from abundant natural resources, a highly attractive candidate for electronic skin.^[118]^ In this context, their attractiveness stems from their ability to serve as either the supporting substrate or an integral component of the functional conductive layer.

**FIGURE 13:**
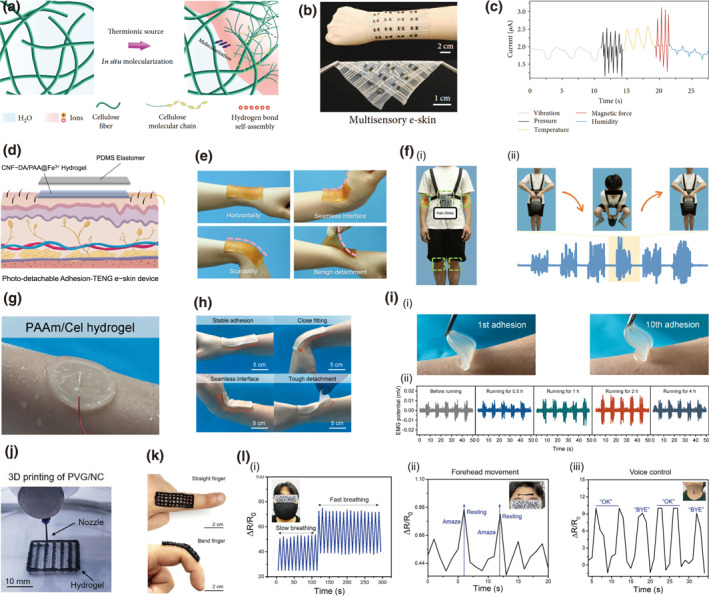
E‐skin applications of cellulose gels. (a–c) Bacterial cellulose ionogel based e‐skin (M‐gel). (a) Schematic of the preparation of M‐gel. (b) Optical images of M‐gel presenting excellent flexibility and adhesion. (c) Multisensory eskin simultaneously sensing the multistimuli. Reproduced with permission.^[17]^ Copyright 2022, AAAS. (d–f) CNF‐DA/PAA@Fe^3+^ self‐powered e‐skin device (Pda‐TENG). (d) Schematic illustration of photo‐detachable PdA‐TENG with a two‐layer structure. (e) Optical images of PdA‐TENG presenting flexibility and adhesion. (f) E‐skin application demonstration of PdA‐TENG: (i) Optical photos of the PdA‐TENG system. (ii) Photos of human motion and corresponding detected signal. Reproduced with permission.^[119]^ Copyright 2024, Springer Nature. (g–i) Sweat‐adaptive adhesive PAAm/Cel hydrogel‐based e‐skin. (PAAm/Cel). (g) Photographs of PAAm/Cel hydrogel applied on sweaty skin. (h) Optical images of PAAM/Cel presenting flexibility and adhesion. (i) E‐skin application demonstration of PAAM/Cel: (i) Adhesion‐detachment performance of PAAm/Cel. (ii) Ambulatory EMG monitoring by PAAm/Cel hydrogel electrodes on sweaty skin after different exercise times. Reproduced with permission.^[120]^ Copyright 2024, Elsevier. (j–l) 3D‐printed e‐skin. (PVG/NC) (j) Photos of 3D‐printing fabrication of PVG/NC. (k) Optical images of PVG/NC presenting flexibility and adhesion. (l) E‐skin application demonstration of PVG/NC: (i) The real‐time breath, (ii) forehead, and (iii) voice control sensing ability of the “E‐skin” patch. Reproduced with permission.^[121]^ Copyright 2025, Wiley‐VCH.

A multifunctional e‐skin is developed by an in situ multiscale molecularization strategy, where a thermionic IL ([Bmim]Cl) is incorporated into a BC hydrogel, controllably constructing an ionogel (M‐gel) (Figure 13a,b).^[17]^ The resulting M‐gel exhibits a multiscale structure with a molecular‐scale H‐bonding network and a nanoscale fiber skeleton, endowing it with remarkable mechanical strength (up to 7.8 MPa), high ionic conductivity (up to 62.58 mS cm^−1^), and rapid self‐healing capability. Inspired by the sensory architecture of the human tongue, the constructed e‐skin integrates various sensing receptors, demonstrating high sensitivity, discriminability, and repeatable responsiveness to multiple stimuli including pressure, touch, temperature, humidity, and magnetic force (Figure 13c).

E‐skins integrated with triboelectric (TENG) or piezoelectric (PENG) nanogenerators can harvest energy from body motion and environmental vibrations, thereby reducing or eliminating their reliance on external batteries for self‐powering.[^122^, ^123^] Leveraging the principle of triboelectric nanogenerators (TENGs) for converting mechanical energy into electrical signals, a self‐powered and photo‐detachable e‐skin (PdA‐TENG) was fabricated by integrating a supramolecular CNF‐DA/PAA@Fe^3+^ hydrogel (Figure 13d).^[119]^ This cellulose gel serves as a conductive and adhesive layer, exhibiting remarkable reversible adhesion and on‐demand, UV‐light‐triggered detachment (Figure 13e). The resulting PdA‐TENG device demonstrated efficient self‐powered capability, generating stable electrical outputs for real‐time monitoring of physiological signals such as blinking and breathing. Furthermore, it supported wireless motion tracking, showcasing its potential for health monitoring and athletic performance assessment (Figure 13f).

The common challenge of adhesion failure in humid environments significantly limits the performance of conventional wearable devices. Designed specifically to overcome this challenge, a poly(acrylamide)/cellulose (PAAm/Cel) hydrogel‐based electronic skin uniquely leverages sweat components to enhance adhesion (Figure 13g,h).^[120]^ The electrolytes in sweat dynamically regulate the internal hydrogen bonds, causing the dissociation of polymer networks and subsequent exposure of abundant polar groups, which significantly strengthens the binding to the skin surface. This sweat‐adaptive hydrogel offers high ionic conductivity (29.8 mS cm^−1^) and achieves strong adhesion on sweating skin, with an interfacial toughness of 268.3 J m^−2^, a tensile strength of 201.7 kPa, and a shear strength of 31.7 kPa. When employed as an electronic skin, PAAm/Cel hydrogel enables high‐quality, stable electromyogram (EMG) signal monitoring during physical exertion, maintaining reliable performance even under continuous sweating conditions (Figure 13i). This makes it ideal for long‐term sports monitoring and rehabilitation tracking.

Demonstrating manufacturing versatility, a PVA/Gelatin/MWCNT/CNCs 3D‐printable e‐skin (PVG/NC) enables the fabrication of customized, multifunctional wearable devices.^[121]^ By leveraging 3D printing for tailored fabrication, the platform enables the creation of customized, multifunctional wearable devices (Figure 13j). PVG/NC exhibits high electrical conductivity (∼5 S m^−1^) and stretchability (∼1000%) (Figure 13k). It supports real‐time monitoring of motion, temperature, and humidity, while also serving as a responsive interface for touch operation and handwriting recognition (Figure 13l), complemented by antibacterial and self‐healing properties.

### 5.4 Flexible energy storage devices

Flexible electronics, which involve fabricating electronic circuits on flexible substrates, are essential for wearable devices and bendable displays. However, traditional electrolytes are fundamentally incompatible with these applications: liquid electrolytes pose leakage and flammability risks in deformable systems, while solid electrolytes typically suffer from high interfacial resistance and rigidity, leading to poor contact and performance degradation under repeated bending or stretching.^[124]^ In contrast, gel electrolytes are ideally suited for flexible electronics. They integrate the high ionic conductivity of liquids with the dimensional stability and enhanced safety of solids, being leak‐proof and capable of suppressing dendrite growth.^[125]^ Crucially, their inherent mechanical flexibility and excellent interfacial compatibility with flexible electrodes enable stable performance under various mechanical deformations, which is vital for robust device design in flexible energy storage systems.

Flexible energy storage systems primarily include advanced supercapacitors and batteries. Their successful operation relies on the synergistic optimization of gel properties, where mechanical robustness ensures structural integrity under deformation. The thermal performance of these devices is intrinsically linked to their overall safety, operational reliability, and the full realization of their inherent performance metrics. Consequently, effective thermal management stands as a foundational consideration in the materials design and system integration for flexible energy storage.​ Supercapacitors are energy storage devices characterized by high power density and rapid charge/discharge kinetics, contrasting with batteries that emphasize high energy density and longer discharge times. Both systems require electrolytes with high ionic conductivity, but their performance priorities lead to distinct demands: supercapacitors necessitate exceptionally high conductivity (typically >10 mS cm^−1^) to enable fast ion transport, coupled with a wide electrochemical window and robust cycle stability for efficient operation at high rates.^[126]^ Batteries, however, focus on electrochemical stability to prevent electrode degradation, dendrite inhibition for safe metal anode cycling, thermal stability under varied conditions, and a high ion transference number to reduce concentration polarization.^[127]^

Derived from renewable resources, cellulose gels are sustainable materials whose hierarchical porous architecture not only enables efficient ion diffusion and electrolyte retention but also provides unique biodegradability and nanochannel‐enhanced ion transport, which are crucial for high‐performance energy storage devices.^[128]^ Their tunable surface functionalization enhances ionic conductivity and mechanical flexibility, supporting applications in flexible supercapacitors and batteries.^[129]^ Derived from abundant and renewable biomass, these gels offer exceptional biodegradability and environmental sustainability, aligning with the principles of circular economy. This unique blend of properties makes cellulose gels a promising electrolyte platform for advancing green and sustainable electrochemical systems.

**FIGURE 14:**
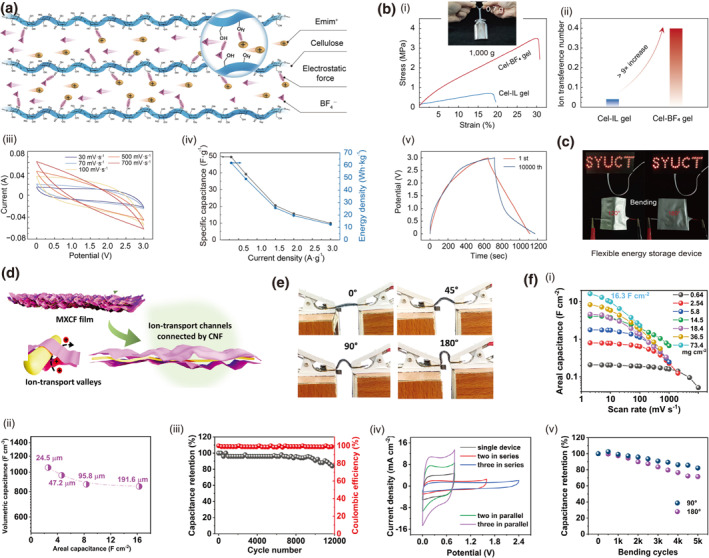
Supercapacitor electrolyte applications of cellulose gels. (a–c) Cellulose ionogel electrode for supercapacitors (Cel‐BF4). (a) Schematic diagram of ion transport in Cel‐BF4 gel. (b) Mechanical and electrochemical properties of Cel‐BF4 gel and Cel‐BF4 gel‐based device: (i) Tensile stress–strain curves showing mechanical reinforcement of Cel‐BF4 gel. (ii) Ion transport reinforcement of Cel‐IL gel. (iii) Cyclic voltammetry curves of the Cel‐BF4 gel‐based device at different scan rates of 0–3 V. (iv) Specific capacitance and energy density curves of the Cel‐BF4 gel‐based device. (v) Cycling test curves of the Cel‐BF4 gel‐based device for 10,000 cycles at 300 mV s^−1^. (c) Digital images of LED light array powered by Cel‐BF4 gel‐based device under bending conditions. Reproduced with permission.^[132]^ Copyright 2025, Wiley‐VCH. (d–f) MXene/CNF hydrogel electrode (MXCF). (d) Schemeatic illustration of ion‐transport valleys in MXCF hydrogel. (e) MXCF wearable device in different bending states. (f) Electrochemical performance of MXCF hydrogel and MXCF device: (i) Areal capacitance of MXCF electrodes. (ii) Volumetric capacitance of different MXCF electrodes. (iii) Stability test of the wearable device after 12,000 charge–discharge cycles at 100 mA cm^−2^ current density. (iv) CV curves of up to three MXCF wearable devices connected by various methods. (v) Retention of device capacitance after 5000 bending cycle tests at different angles. Copyright 2024, Wiley‐VCH.

The application of cellulose gels as electrolytes in supercapacitors has demonstrated significant improvements in both energy density and cycling stability, with numerous research examples providing strong evidence for these advancements.[^130^, ^131^] A high‐performance cellulose ionogel (Cel‐BF4), comprising cellulose and 1‐ethyl‐3‐methylimidazolium tetrafluoroborate ([Emim]BF4), is engineered through molecular network reconstruction.^[132]^ This strategy enhances hydrogen bonding for mechanical robustness (tensile strength 3.5 MPa) while reducing electrostatic interactions to achieve high ionic conductivity (14.3 mS cm^−1^) and a wide voltage window (3.0 V) (Figure 14a). In an integrated supercapacitor configuration, where the gel functions as both electrolyte and separator with embedded active materials (activated carbon, MoS_2_, MWCNTs), the device delivers an exceptional energy density exceeding 60 Wh kg^−1^ along with outstanding cycling stability, retaining over 97% capacitance after 10,000 cycles (Figure 14b,c). These properties make it particularly suitable for flexible energy storage applications requiring both mechanical durability and electrochemical performance.

To address the trade‐off between areal and volumetric capacitance, a MXene/CNF hydrogel electrode was engineered through electric‐field‐guided co‐assembly and dehydration‐induced collapse.^[133]^ This process creates an interwoven porous network where crumpled MXene sheets generate intersheet porosity while CNFs act as spacers and connective bridges, forming interconnected ion transport channels across multiple length scales (Figure 14d). When employed as a gel electrolyte, the material exhibits an ionic conductivity of 14.3 mS cm^−1^ and a wide electrochemical stability window of 3.0 V, while maintaining remarkable mechanical flexibility and performance stability under repeated bending deformation (Figure 14e). Owing to these properties, the assembled supercapacitor achieves a high energy density of 61.87 Wh kg^−1^ while maintaining 97% capacitance retention over 10,000 cycles (Figure 14f).

**FIGURE 15:**
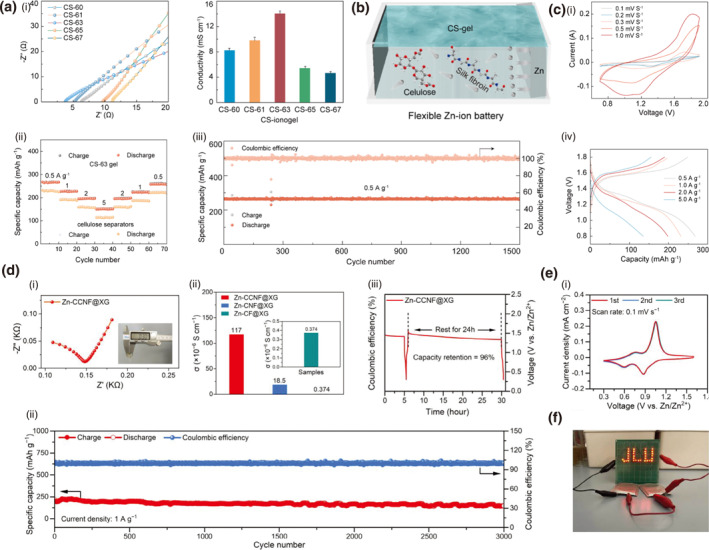
Battery electrolyte applications of cellulose gels. (a–c) Cellulose and silk fibroin based supramolecular dual‐network gel electrolyte (CS‐gel). (a) Electrochemical properties of CS‐gels: (i) Electrochemical impedance spectroscopy (EIS) curves of various CS‐gels. (ii) Ionic conductivity of CS‐gels. (b) Schematic illustration of compositions of CS‐gels. (c) Electrochemical properties of CS‐gel battery electrolytes: (i) Cyclic voltammetry (CV) curves of the battery. (ii) Specific capacities of two flexible zinc‐ion batteries based on CS63 gel and cellulose separator. (iii) Cycling performance tests under different bending angles. (iv) Galvanostatic charge‐discharge (GCD) curves of the battery. Reproduced with permission.^[135]^ Copyright 2025, Elsevier. (d–f) Carboxylated cellulose nanofibrils based solid‐state electrolyte (Zn‐CCNF@XG). (d) Electrochemical properties of Zn‐CCNF@XGs: (i) EIS spectra of Zn‐CCNF@XG. (ii) Zn^2+^ transference numbers of Zn‐CNF@XG and Zn‐CCNF@XG. (iii) Columbic efficiency of Zn‐CCNF@XG. (e) Electrochemical properties of Zn||NVO full battery: (i) CV curves for NVO electrode test. (ii) Cycling stability of Zn||NVO typical coin battery at a current density of 1 A g^−1^. (f) Optical photo of two assembled pouch batteries in series powered for a LED flat panel. Reproduced with permission.^[136]^ Copyright 2024, Wiley‐VCH.

In rechargeable batteries, cellulose gel electrolytes effectively address critical challenges including dendrite suppression and wide‐temperature operation.^[134]^ A supramolecular dual‐network gel electrolyte (CS‐gel), composed of cellulose and silk fibroin, is developed for flexible Zn‐ion batteries.^[135]^ This bio‐based gel is designed by co‐dissolving cellulose and silk fibroin in a green DES, followed by ethanol‐induced assembly. This mild process preserves SF's inherent ability to form functional β‐sheet domains, which interweave with cellulose's H‐bond network to create a synergistic supramolecular dual‐network structure. The CS‐gel demonstrates a robust tensile strength of 1.14 MPa and a high ionic conductivity of 14.39 mS cm^−1^ (Figure 15a). In flexible Zn//MnO_2_ batteries, it ensures outstanding cycling stability with 97.67% capacity retention after 1500 cycles, along with reliable performance under bending, demonstrating great potential for flexible electronics (Figure 15b,c).

A nanoengineered functional solid‐state electrolyte, Zn‐CCNF@XG, is developed from carboxylated cellulose nanofibrils coordinated with Zn^2+^ for high‐performance dendrite‐free all‐solid‐state zinc‐ion batteries.^[136]^ The design utilizes carboxyl and hydroxyl groups as Zn^2+^ hopping sites, aiming to construct rapid ion transport channels. This approach, involving TEMPO‐oxidation and nano‐engineering, significantly reduces the dissociation energy for Zn^2+^ desolvation, enabling the electrolyte to achieve a high ionic conductivity of 1.17 × 10^−4^ S cm^−1^ and a superior Zn^2+^ transference number of 0.78 (Figure 15d). When deployed in Zn||NaV_3_O_8_·1.5H_2_O full cells, the electrolyte ensures exceptional cycling stability, retaining 83.46% capacity after 3000 cycles at 1 A g^−1^ with a near‐perfect Coulombic efficiency of 99.99%, effectively suppressing zinc dendrites and hydrogen evolution (Figure 15e,f).

### 5.5 Remote interaction and artificial intelligence applications

Hydrogels are increasingly utilized in HMI due to their inherent softness, stretchability, and biocompatibility. To meet these demanding application scenarios, conductive polymer‐based hydrogels (CPHs) have emerged as a promising sensing platform.^[137]^ Their functionality primarily relies on the piezoresistive effect, where mechanical deformations (strain or pressure) alter the internal conductive network, leading to measurable resistance changes.^[138]^ This mechanism underpins high sensitivity and fast response. Engineered with exceptional mechanical robustness, environmental stability, and reliable adhesion, CPHs provide the foundation for high‐fidelity physiological monitoring. These critical attributes facilitate their deployment in advanced interactive systems, including smart gloves and implantable bioelectronic interfaces, thereby highlighting the considerable promise of CPHs for next‐generation HMI. The self‐powering capabilities of hydrogels through energy harvesting mechanisms like triboelectric or piezoelectric effects further enhance their potential for autonomous, battery‐free HMI devices, promoting sustainability and long‐term usability.^[139]^

**FIGURE 16:**
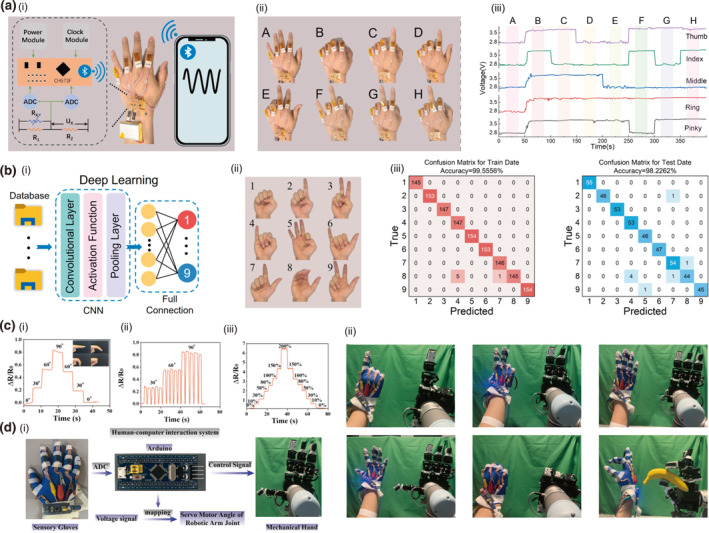
Remote interaction and AI applications of cellulose gels. (a, b) MXene/CNF pressure sensors: (a) A flexible wireless feedback system composed of MXene/CNF pressure sensors: (i) Modules of flexible wireless feedback system. (ii) Images of hand gestures and (iii) Corresponding voltage response signals. (b) Deep learning for gesture recognition: (i) Schematic diagram of deep learning system. (ii) Images of hand gestures. (iii) Confusion matrix diagram of the training set and test set. Reproduced with permission.^[140]^ Copyright 2025, Elsevier. (c, d) HLAI ionogel based strain sensors: (c) Strain sensing performance of HLAI ionogels: (i) Δ*R*/*R*_0_ of different bending angles. (ii) Δ*R*/*R*_0_ of different bending angles under 5 cycles. (iii) Δ*R*/*R*_0_ of different strains. (d) (i) Photos of human‐computer interaction system and (ii) Sensory gloves controlling mechanical hand. Reproduced with permission.^[141]^ Copyright 2024, Elsevier.

In practical implementations, hydrogel‐based sensors are increasingly being integrated with advanced technologies to enable sophisticated remote and real‐time interactions. A porous, flexible pressure‐sensitive layer composed of MXene and cellulose nanofiber (CNF) forms a composite gel with a patchy‐protrusion and porous structure. For human‐computer interaction, the gel was integrated into a miniaturized flexible wireless feedback system and successfully captured distinct voltage signals corresponding to various finger gestures (Figure 16a). The transmitted data served as input for a deep learning framework utilizing a convolutional neural network (CNN), which achieved a high recognition accuracy of 98.22% for nine distinct gestures (Figure 16b).^[140]^ In another approach, a novel solvent‐free ionic gel (HLAI) composed of hydroxypropyl cellulose (HPC), lipoic acid (LA), acrylic acid (AA), and [C_2_VIm]Br IL forms a multi‐stage network stabilized by dense hydrogen bonding and dynamic disulfide bonds. The gel exhibited excellent sensing performance, reliably monitoring finger joint movements by generating distinct and stable resistance changes (Δ*R*/*R*_0_) (Figure 16c). For advanced human‐computer interaction, the gel was integrated into a sensory glove to precisely control a robotic arm, enabling it to accurately mimic complex human gestures (Figure 16d).^[141]^ These systems are proved to be highly suitable for flexible electronics and continuous health monitoring, thereby underscoring the transformative role of hydrogels in enabling next‐generation remote HMI solutions.

## 6 SUMMARY AND PROSPECT

Looking forward, cellulose gels have garnered significant attention due to their exceptional mechanical robustness, biocompatibility, and versatile tunability, positioning them as a leading material for sustainable technologies. Their potential spans a wide spectrum of advanced applications, from next‐generation biomedical engineering to flexible electronics and energy storage devices. However, the broader development and commercial translation of these promising materials face significant challenges.[^142^, ^143^, ^144^, ^145^] Further development of cellulose gels is constrained by the following reasons:

(1) The large‐scale utilization of cellulose is hindered by challenges in dissolution, mainly due to the lack of viable green solvent systems that are cost‐effective and easy to process. Representative solvents, such as ILs, require complex synthesis and remain expensive. Although DESs are more affordable, their cellulose solubility is generally limited. These limitations in solvent systems impede the scaling‐up of advanced gel synthesis from laboratory research to commercial production, consequently restricting the industrial application of cellulose‐based functional materials.
(2) Cellulose gel electrolytes face significant performance challenges, particularly in low‐temperature environments. Commercial supercapacitor and battery electrolytes must maintain high ionic conductivity, mechanical stability, and safety across wide temperature ranges (−40°C–60°C). However, cellulose gels often underperform under subzero conditions due to material‐specific limitations: hydrogels suffer from ice crystal formation, ion gels struggle with high cost and intricate conductivity‐stability trade‐offs, while aerogels exhibit mechanical fragility and poor interfacial contact. Although modifications like inorganic ion incorporation (e.g., Li^+^, Ca^2+^) or dual‐network designs can enhance frost resistance, most cellulose gels still require further optimization to match the durability and broad operational range of conventional electrolytes.
(3) Cellulose gels face a challenge in balancing strength, toughness, and fatigue resistance for flexible robotics, owing to an inherent trade‐off in their network structure between energy dissipation and elastic recovery. For example, increasing crosslinking density to enhance strength often induces embrittlement, whereas improving toughness may compromise strength and cause instability under cyclic loading. This trade‐off obstructs the development of materials that are strong, deformable, and durable simultaneously. Additionally, the inherent hydrophilicity of cellulose causes water absorption in humid environments, which plasticizes the polymer chains and leads to a degradation of mechanical performance over time, thus undermining long‐term reliability.

Therefore, to address these hurdles, future research will focus on multidisciplinary efforts that combine advanced material design with innovative processing techniques. The overarching goal is to develop integrated strategies to enhance the overall performance and versatility of cellulose gels, thereby unlocking their full potential for practical applications.

(1) Development of sustainable solvents: Future breakthroughs are likely to hinge on the design of novel, recyclable, and cost‐effective DESs.^[135]^ The core scientific challenge lies in unraveling the structure‐property relationships between the hydrogen bond donor/acceptor structures and their cellulose dissolution capacity and dissolution kinetics. Guiding solvent design through high‐throughput screening and molecular simulations represents a critical pathway toward achieving scalable applications.
(2) Multi‐scale Mechanical Stabilization:​ Advancing cellulose gels necessitates coordinated strategies across molecular, supramolecular, and macroscopic scales. Molecular‐level functional group modification, tunable supramolecular networks, and hierarchical structural alignment work synergistically to enhance mechanical strength, toughness, and environmental stability. Future efforts should focus on the integrated design of these multi‐scale approaches to achieve optimal performance in demanding applications.
(3) Design for thermal stability performance​: For cellulose gel electrolytes in supercapacitors and batteries, achieving reliable performance across extreme temperatures is paramount. Molecular‐scale strategies such as intelligent ion coordination and dynamic bond engineering are essential to overcome the conductivity‐mechanical robustness trade‐off under thermal stress. Establishing robust structure‐property relationships will ensure operational stability in real‐world thermally fluctuating environments.
(4) Actuation‐response and motion enhancement:​ For flexible robotics, future cellulose gel development must address the fundamental trade‐offs among strength, toughness, and fatigue resistance by rationally designing hydrogen bond networks to regulate energy dissipation. Concurrently, improving stimuli‐responsive performance requires synergistic approaches, including precise molecular modification of cellulose to tailor interaction dynamics and macroscopic structural alignment for efficient, directional actuation. To accelerate this development, combining computational materials science for network design with soft robotics for functional integration will be crucial to bridge molecular optimization and application‐oriented performance.

Harnessing the full potential of cellulose gels will unlock a new era of sustainable advanced materials. Their inherent versatility positions them as foundational components for groundbreaking applications in green electronics, adaptive flexible robotics, and personalized biomedicine. This progress will not only drive technological innovation but also make significant contributions towards a more sustainable and resource‐efficient future.

## AUTHOR CONTRIBUTIONS

Dawei Zhao supervised the project. Zeshi Li wrote the paper and drew schematic diagrams. Geyuan Jiang and Jianhong Zhou were involved in the collection of topic literature and the organization of pictures. Weihua Zhang, Donghan Li and Dawei Zhao made the guidance amendments to the pictures and revised the paper. All authors have read and approved the final submitted manuscript.

## CONFLICT OF INTEREST STATEMENT

The authors declare no conflicts of interest.

## Data Availability

The data that support the findings of this study are available on request from the corresponding author. The data are not publicly available due to privacy or ethical restrictions.
